# Dual targeting of mTOR/IL-17A and autophagy by fisetin alleviates psoriasis-like skin inflammation

**DOI:** 10.3389/fimmu.2022.1075804

**Published:** 2023-01-18

**Authors:** Tithi Roy, Sergette Banang-Mbeumi, Samuel T. Boateng, Emmanuelle M. Ruiz, Roxane-Cherille N. Chamcheu, Lin Kang, Judy A. King, Anthony L. Walker, Bolni Marius Nagalo, Konstantin G. Kousoulas, Stephane Esnault, Shile Huang, Jean Christopher Chamcheu

**Affiliations:** ^1^ School of Basic Pharmaceutical and Toxicological Sciences, College of Pharmacy, University of Louisiana at Monroe, Monroe, LA, United States; ^2^ School of Nursing and Allied Health Sciences, Louisiana Delta Community College, Monroe, LA, United States; ^3^ Department of Pathobiological Sciences, School of Veterinary Medicine, Louisiana State University, Baton Rouge, LA, United States; ^4^ Biomedical Research, Edward Via College of Osteopathic Medicine, Monroe, LA, United States; ^5^ Department of Biomedical Sciences and Pathobiology, Virginia-Maryland College of Veterinary Medicine, Virginia Tech, Blacksburg, VA, United States; ^6^ Department of Pathology and Translational Pathobiology, Louisiana State University Health Sciences Center, Shreveport, LA, United States; ^7^ School of Clinical Sciences, College of Pharmacy, University of Louisiana at Monroe, Monroe, LA, United States; ^8^ Department of Pathology, University of Arkansas for Medical Sciences (UAMS), Little Rock, AR, United States; ^9^ The Winthrop P. Rockefeller Cancer Institute, UAMS, Little Rock, AR, United States; ^10^ Division of Allergy, Pulmonary and Critical Care Medicine, Department of Medicine, School of Medicine and Public Health, Madison, WI, United States; ^11^ Department of Biochemistry and Molecular Biology, Louisiana State University Health Sciences Center, Shreveport, LA, United States; ^12^ Department of Hematology and Oncology, Louisiana State University Health Sciences Center, Shreveport, LA, United States; ^13^ Feist-Weiller Cancer Center, Louisiana State University Health Sciences Center, Shreveport, LA, United States

**Keywords:** autophagy, fisetin, psoriasis, Akt/mTOR and IL-17A, rapamycin, topical administration, psoriasis-like skin inflammation, keratinocytes RNA-sequencing

## Abstract

Psoriasis is a chronic autoimmune inflammatory skin disorder characterized by epidermal hyperplasia and aberrant immune response. In addition to aberrant cytokine production, psoriasis is associated with activation of the Akt/mTOR pathway. mTOR/S6K1 regulates T-lymphocyte activation and migration, keratinocytes proliferation and is upregulated in psoriatic lesions. Several drugs that target Th1/Th17 cytokines or their receptors have been approved for treating psoriasis in humans with variable results necessitating improved therapies. Fisetin, a natural dietary polyphenol with anti-oxidant and anti-proliferative properties, covalently binds mTOR/S6K1. The effects of fisetin on psoriasis and its underlying mechanisms have not been clearly defined. Here, we evaluated the immunomodulatory effects of fisetin on Th1/Th17-cytokine-activated adult human epidermal keratinocytes (HEKa) and anti-CD3/CD28-stimulated inflammatory CD4^+^ T cells and compared these activities with those of rapamycin (an mTOR inhibitor). Transcriptomic analysis of HEKa revealed 12,713 differentially expressed genes (DEGs) in the fisetin-treated group compared to 7,374 DEGs in the rapamycin-treated group, both individually compared to a cytokine treated group. Gene ontology analysis revealed enriched functional groups related to PI3K/Akt/mTOR signaling pathways, psoriasis, and epidermal development. Using *in silico* molecular modeling, we observed a high binding affinity of fisetin to IL-17A. *In vitro*, fisetin significantly inhibited mTOR activity, increased the expression of autophagy markers LC3A/B and Atg5 in HEKa cells and suppressed the secretion of IL-17A by activated CD4^+^ T lymphocytes or T lymphocytes co-cultured with HEKa. Topical administration of fisetin in an imiquimod (IMQ)-induced mouse psoriasis model exhibited a better effect than rapamycin in reducing psoriasis-like inflammation and Akt/mTOR phosphorylation and promoting keratinocyte differentiation and autophagy in mice skin lesions. Fisetin also significantly inhibited T-lymphocytes and F4/80^+^ macrophage infiltration into skin. We conclude that fisetin potently inhibits IL-17A and the Akt/mTOR pathway and promotes keratinocyte differentiation and autophagy to alleviate IMQ-induced psoriasis-like disease in mice. Altogether, our findings suggest fisetin as a potential treatment for psoriasis and possibly other inflammatory skin diseases.

## Introduction

Psoriasis is a common chronic autoimmune inflammatory skin disease that affects more than 125 million people worldwide, including 7.55 million afflicted Americans with similar prevalence among males and females (1, 2). Clinically, psoriatic skin lesions are characterized by uncontrolled epidermal hyperplasia, aberrant differentiation, dysregulated cytokine network signaling, and infiltration of immune cells into the epidermis and dermis (3, 4).

Activated and differentiated helper T cell subsets (Th1, Th17, and Th22) and their effector pro-inflammatory cytokine (e.g. TNFα and IL-17A) mediators play a major role in the pathophysiology of psoriasis, promote the synthesis of acute phase proteins and are often associated with extracutaneous and systemic comorbidities (5–8). They direct local tissue inflammation by driving activated keratinocytes and myeloid lineage cells to release proinflammatory cytokines and neutrophil-attracting chemokines that maintain chronicity (9). The expression of these mediators by CD4^+^ T lymphocytes is regulated by *nuclear factor kappa B* (NF-κB) and signal transducer and activator of transcription 3 (STAT3) signaling pathways (10–12). Increased tumor necrosis factor-alpha (TNF-α) produced by dendritic cells and other activated immune cells and nitric oxide (NO) generated by keratinocytes, assist naïve helper T cells to differentiate into Th1 and Th17 subsets, which are important mediators of psoriatic hyperplasia and inflammation (13).

Recent studies have shown that the PI3K-Akt-mTOR signaling pathway that is important in cell metabolism, growth, and survival is dysregulated and linked to the pathophysiology of diverse skin diseases including psoriasis (14–17). The activation of this pathway contributes to increased secretion of Th1/Th17 mediators (14), including IL-1, IL-17E (IL-25) and IL-23, which initiate the proliferation of Th17 cells to increase psoriatic inflammation and keratinocyte hyperplasia, while recruiting neutrophils and secreting vascular endothelial growth factor (VEGF) (18–22). Moreover, the interleukin (IL)-6 also used herein is a cytokine with a pleotropic immune-related activity. IL-6 and/or IL-22 have pro-inflammatory activities toward lymphoid and myeloid lineage immune cells, and keratinocytes, which along with other cytokines drive their proliferation, differentiation and acute phase response observed in psoriasis (23). For instance, in the presence of TGF-ß and IL-6, naive T cells differentiate into Th17 cells, producing effectors that drive psoriasis immunopathogenesis (24).

Currently, there is no cure for psoriasis. Recently, a number of biologics targeting Th1 and Th17 cytokines or their receptors (25–28) were developed as anti-psoriasis options. These clinically available anti-psoriatic agents block immune cell activation and hence control the inflammatory response and epidermal hyperplasia (28). They are effective for patients with moderate-to-severe psoriasis at an early stage, but are not particularly useful for most (80-90%) patients with mild-to-moderate disease due to high cost, inaccessibility, off-target effects, and potential side effects related to prolonged administration (28). Therefore, cost-effective and non-toxic therapies that can efficiently modulate multiple biomarkers to achieve long-term benefits for psoriasis patients are still needed. Additionally, targeting mTOR/effectors and autophagy to modulate keratinocytes and T-lymphocyte activation would curtail the secretion of cytokines to clinically decrease inflammation and epidermal hyperplasia.

We identified fisetin (3,7,3’,4’-tetrahydroxyflavone), a naturally occurring dietary small molecule abundant in colored fruits and vegetables. We and others have shown that fisetin exhibits diverse properties including anti-angiogenic, anti-oxidant, and anti-inflammatory actions (29, 30). Fisetin binds directly to the p70 ribosomal protein S6 kinase 1 (p70S6K1) and mammalian target of rapamycin (mTOR) with remarkable affinity values of 940 nmol and 11000 nmol, respectively, and inhibits their activity (31). Fisetin significantly suppresses dermal immune cell infiltration including eosinophils, mast cells, and CD4^+^ and CD8^+^ T-cells (32, 33). Additionally, fisetin suppresses the production of cytokines and chemokines such as interferon-gamma (IFN-γ) and interleukin-4 (IL-4) by activated CD4^+^ T cells, reduces IL-31 by stimulated mast cells, and enhances the production of the anti-inflammatory cytokine, IL-10 in atopic dermatitis-like (AD) mouse models (32, 33). Moreover, fisetin inhibits the release of proinflammatory cytokines by activated peripheral blood mononuclear cells (PBMCs) and keratinocytes (34), and the activation of PI3K/Akt/mTOR/MAPK in *in vitro* models of psoriasis (31, 34). We hypothesized that fisetin could effectively inhibit T-cell activation and improve keratinocyte functions to reduce features observed in psoriasis. Here we tested the ability of fisetin to inhibit *1)* IL-17A production by human CD4^+^ T lymphocytes, both activated and co-cultured with human keratinocytes *in vitro*, and *2)* cytokine (IL-17A and TNFα)-induced responses in keratinocytes. We also validated the ability of topical fisetin administration to improve psoriasiform dermatitis *in vivo* in a well-established imiquimod (IMQ) psoriasis mouse model. We present pre-clinical evidence for fisetin as a potential treatment for psoriasis.

## Results

### Fisetin treatment inhibits IL-17A plus TNF-α-induced keratinocyte related inflammatory responses

**Figure 1:**
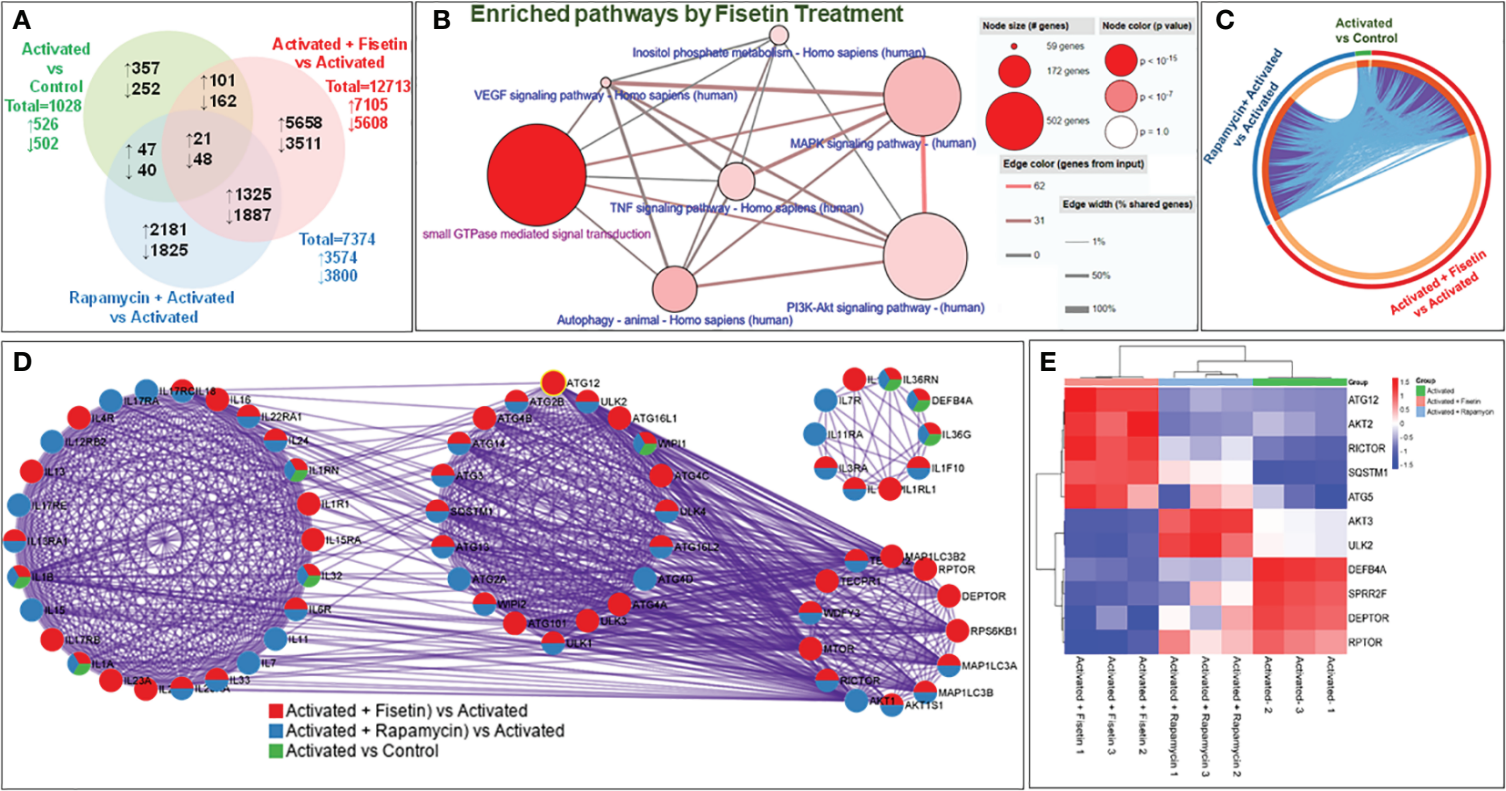
Fisetin treatment modulates several targets and signaling pathways in IL-17A/TNFα-stimulated primary keratinocytes. **(A)** Venn diagram showing the genes that were differentially regulated with >2-fold increase (shown as upward arrow) or decrease (downward arrow) from each comparison across the different groups of treatment and activation with cytokines in keratinocytes. **(B)** Metascape analysis at systems level of transcriptome profiles of 3 groups in circus plot: Activated vs Control (green), Activated + Fisetin vs Activated (red) and Activated + Rapamycin vs Activated (blue) *in vitro*. The plot shows how 1.5-fold changes differentially expressed genes are overlapped: Each arc represents as individual gene. Dark orange color represents the genes shared by multiple groups which forms purple link. On the contrary, the light orange arc represents unique gene to that list from the group. Light blue color represents different genes but shared ontology terms. There is a trend to see more common functional overlap that means subset of genes of the same biological process. **(C)** Differential enrichment analysis illustrates the transcriptomic enrichment analysis of genes involved in different important ontology terms (GO/KEGG) in cytokine-activated keratinocytes compared with control represented by circles. Enrichment study of GO/KEGG and hall mark gene sets. The overlap between the input gene lists increases with the thickness of pink linkages. The detailed enrichment study is shown in **Figure S1** online. **(D)** All input gene lists were also merged into one list and resulted in a PPI (Protein-Protein Interaction) network. Network nodes are displayed as pies. Color code for pie sector represents a gene list and is consistent with the colors used for Activated vs Control (green), Activated + Fisetin vs Activated (red) and Activated + Rapamycin vs Activated (blue). To identify neighborhoods where proteins are densely connected, the MCODE algorithm was used to this network with biological meaning, which shows that a cytokine mediated signaling, cellular response to cytokine stimulus and autophagosome organization are related to transcriptome expression in the treatment group differentially. **(E)** The heatmap shows differential expression of mTOR pathway components and autophagy related transcriptome in each replicate of 3 groups. Overlaps at the gene level, where purple curves link identical genes in three groups, including the shared term level, where blue curves link genes that belong to the same enriched ontology term are shown in a circular 3 plot **(C)**.

RNA-Seq analysis of HEKa preincubated with or without fisetin and/or subsequently stimulated with TNF-α/IL-17A found 1,028 differentially expressed genes (DEGs) between HEKa (Control) and HEKa activated with TNF/IL-17A (Activated), including 526 upregulated genes and 502 downregulated genes. Treatment with fisetin (15 μM) resulted in a total of 12713 DEGs, including 7105 upregulated genes and 5608 downregulated genes, whereas rapamycin (100 nM) treatment led to 7374 DEGs, comprising 3574 upregulated genes and 3800 downregulated genes (GEO accession # GSE217552). There are 122 differentially upregulated and 210 differentially downregulated genes in common between the activated + fisetin and the activated group alone (**Figures 1A**, **S2A**), notably many psoriasis-associated genes such as *CCL20*, *IL36G*, *IL23A* and *NFKBIZ.*

To understand the pathways affected by those DEGs in the fisetin-treated group compared to the activated group, we conducted an overexpression analysis of the Consensus PathDB database (35). This provided explorable molecular interaction data in the GO terms, the KEGG pathway database, and the Reactome database. Fisetin treatment increased the number of effectors associated with the MAPK signaling pathway, the TNF signaling pathway, the small GTPase mediated signal transduction pathway, the inositol phosphate metabolism pathway, autophagy, and the VEGF signaling system, which are all known to be dysregulated or involved in psoriasis (**Figure 1B**). Overlapping DEGs when the three groups (activated vs Control, activated + fisetin vs activated, and activated + Rapamycin vs activated) were analyzed by Metascape (**Figures 1C, D**) (36).

Treatment with fisetin resulted in alterations in 211 out of 354 genes identified in the PI3K-Akt signaling pathway, 392 out of 502 genes belonging to the small GTPase mediated signaling pathway, and 190 out of 294 in the MAPK signaling pathway. Differential enrichment of 27 out of 40 genes shared by both the PI3K-Akt signaling pathway and small GTPase-mediated signal transduction was observed in activated + fisetin treated keratinocytes (**Figure 1C** and **Supplementary file: S1** highlighted).

Genes downregulated by fisetin-treatment were analyzed by GO ontology and KEGG and changed pathways included GTPase activators (130 genes, padj 3.78E^-06^) and regulators (141 genes, padj 2.64E^-06^), Ras signal transduction (188 genes, padj 1.15E^-05^), cellular components of cell leading edge, actin cytoskeleton (80 genes, padj 0.019937304), VEGF signaling (33 genes, padj 0.003428597), phospholipase D signaling (59 genes, padj 0.014053746), and TNF signaling (45 genes, padj 0.046010541). Fisetin also downregulated the molecular functions of protein binding and signal transduction carried out by Rho, Rab, and Ras. In addition to upregulating mRNA processing (229 genes, padj 1.54E^-13^), fisetin treatment also upregulated autophagy (183 genes, 6.21E^-23^), myeloid leukocyte-mediated immunity (194 genes, 0.016782866), protein targeting endoplasmic reticulum (84 genes), and other pathways [**Figure S1B**; **Supplementary File_S_1** (highlights)]. A summary of 20 pathways that were either upregulated or downregulated as a result of treatment with fisetin is illustrated in **Figure S1**.

Next, the MCODE algorithm of Cytoscape was applied to the network to find more interconnected neighborhoods where proteins are densely connected using all the DEGs employed as inputs. Significant modules of protein-protein interaction (PPI) network by gene count among the 3 different groups as depicted in the circular layout (**Figure 1D**). This PPI analysis disclosed genes involved in mTOR pathway, autophagy, cellular response to cytokine stimulus, cytokine mediated signaling pathway and inflammatory response in the activated + fisetin treated group. In activated vs control group, inflammatory response genes (GO:0006954), inflammatory response to antigenic stimulus (GO:0002437) and cellular response to lipopolysaccharide (GO:0071222) were found as they are related to inflammatory response in keratinocytes. It was also observed that fisetin or rapamycin treatment modulated activation-induced genes related to mTOR pathway and autophagy (see **Figures 1B, D**). Additionally, fisetin treatment differentially modulated the mTOR pathway related genes such as Raptor, Rictor and Deptor, which are core genes of the mTORC1 and mTORC2 complexes, compared to TNFα plus IL-17A alone (activated) group. A similar trend was observed in activated + rapamycin treated group. Furthermore, skin related genes such as antimicrobial peptide, defensin beta 4A (*DEFB4A*), was upregulated in the TNFα plus IL-17A activated group, but was significantly downregulated by fisetin treatment when compared to rapamycin.

In psoriasis, proline rich crosslink envelope protein (SPRR) and late cornified envelope proteins (LCEs) are tightly regulated during keratinocyte terminal differentiation, while the transglutaminase substrates for LCE formation are downregulated (37, 38). Herein, we also observed differential regulation of SPRR gene isoforms, while most of the LCEs gene isoforms were upregulated in keratinocytes upon fisetin treatment when compared to activated vs control cells. Since over 31 autophagy-related gene (ATG) products have been identified to regulate autophagy (39), and rapamycin has been shown to induce autophagy (40), a transcriptomic correlation heatmap analysis was performed using the average clustering scheme. It was identified that fisetin and rapamycin individually induced the expression of *ATG5*, *ATG12, SQSTM1*, and *ULK2* (**Figure 1E**).

### Fisetin downregulates cytokine-induced keratinocyte responses by inhibiting the mTOR-Akt pathway

**Figure 2:**
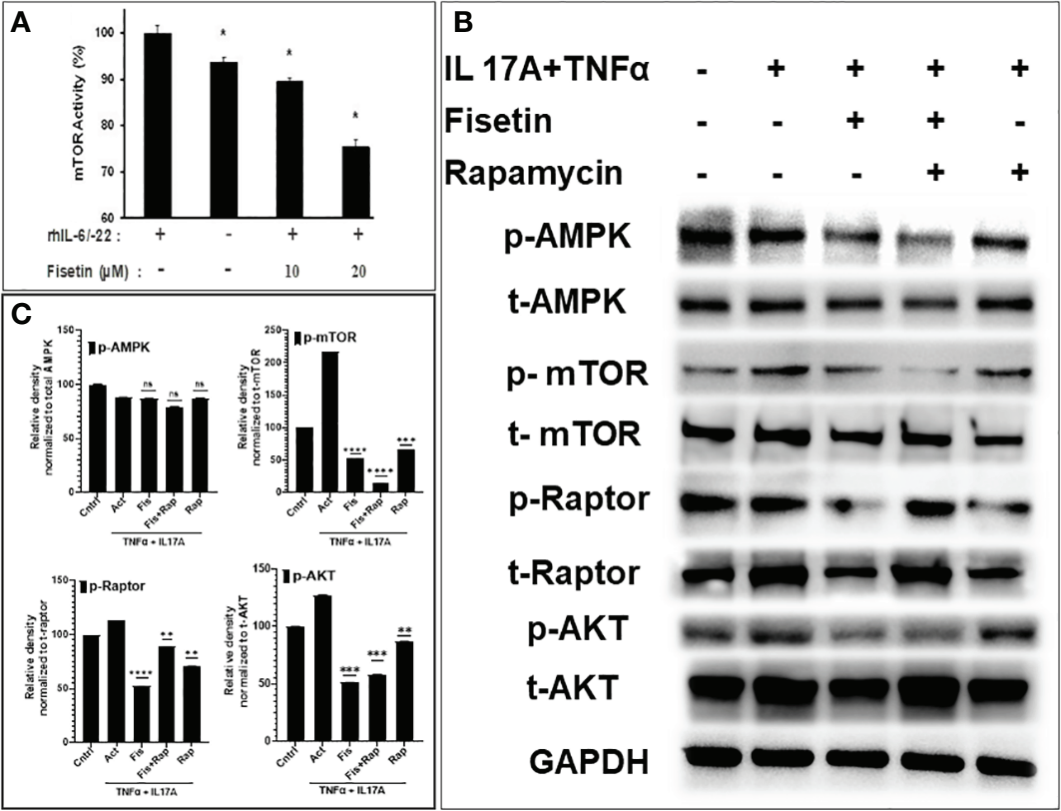
Fisetin suppresses mTOR kinase activity and reduces phosphorylated mTOR pathway components in cytokine-activated human epidermal keratinocytes. **(A)** ELISA analysis of mTOR kinase activity in control, fisetin-treated, or cytokine [rhIL-6/IL-22 (15ng/ml each)]-treated primary keratinocytes lysates. **(B)** Western blot analysis of differential protein expression levels of p-Akt^Ser473^, p-AMPK (Thr^172^)?, p-mTOR^Ser2448^, and p-raptor (Ser^792^) in fisetin (15µM) treated, rapamycin (100nM) or (IL-17A+TNFα)-activated HEKa compared with untreated control cells. **(C)** Graphs of the semi-quantified intensity of bands for p-Akt, p-AMPK, p-mTOR, and p-raptor. Bar represents means ± SD of three separate experiments each performed in triplicate. *P < 0.05, **P < 0.01, ***P < 0.001, and ****P < 0.0001 vs. control for IL-6/IL-22-treated only or vs. IL-22 or IL-17A+TNFα for fisetin-treated cells. ns, not significant.

To discover the mechanism by which fisetin inhibits the pro-inflammatory processes associated with keratinocyte differentiation, primary keratinocytes pretreated with different concentrations of fisetin (10-20µM), and activated with or without 20 ng/mL each of rh IL-6 and rhIL-22 were analyzed for mTOR activity. ELISA analysis of cell lysates showed a dramatic increase in the activity of mTOR upon activation with IL-6 plus IL-22 compared with the non-activated control group. Fisetin pretreatment significantly reduced cytokine (IL-6 plus IL-22)-induced activation of mTOR activity in a dose-dependent manner (**Figure 2A**). We and others previously demonstrated that IL-22 induces keratinocyte proliferation, which can be suppressed by fisetin through blocked of the central mTOR pathway (34, 41). The combination of cytokines (TNFα and IL-17A) both being important targets in psoriasis development were utilized *in vitro* to activate and promote keratinocytes proliferation and inflammatory responses while examining the intervention with the test compounds. Treatment with fisetin reduced cytokine-induced activity of mTOR in keratinocytes (**Figure 2**). Since our transcriptomic data showed that fisetin-treatment differentially modulated mTOR, its effectors, and autophagy in IL-17A plus TNFα activated keratinocytes, we next examined their effects on protein expression levels of the different mTOR pathway and autophagy components. We observed that compared to non-activation (control), the combination of cytokines (TNFα and IL-17A) stimulation resulted in the activation of proteins associated with the Akt/mTOR pathways in keratinocytes. Correspondingly, treatment with fisetin or rapamycin resulted in significant decrease in the protein phosphorylation levels of Akt, mTOR, and Raptor, with the effects being more pronounced in fisetin compared to rapamycin group (**Figures 2B, C**). However, fisetin or rapamycin had no effect on the phosphorylation of AMPK when measured in relation to total AMPK (see **Figures 2B, C**).

### Fisetin induces autophagy in TNF-α/IL-17A-activated keratinocytes *via* upregulation of autophagic markers

**Figure 3:**
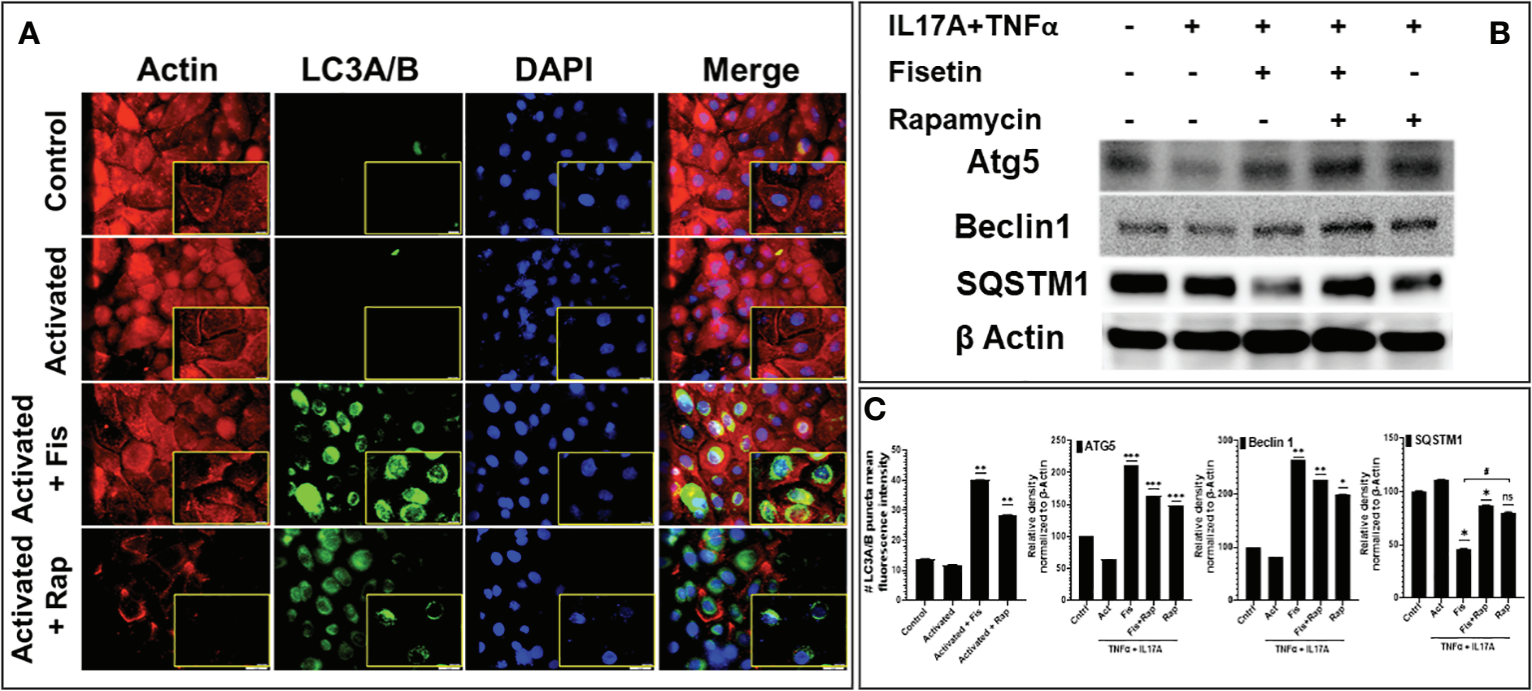
Fisetin induces the expression of autophagy markers in cytokine-stimulated primary epidermal keratinocytes. **(A)** Representative photographs of the immunofluorescence staining for actin (red) and LC3A/B (green) and the DAPI staining (blue; for nucleus) in fisetin (15 µM), rapamycin (100 nM) or IL-17A+TNFa)-treated HEKa compared with inactivated control keratinocytes/HEKa. **(B)** Western blot analysis for differential protein levels of autophagy markers Atg5, Beclin1 and SQSTM1 in fisetin- or rapamycin-treated primary keratinocytes activated with cytokines (IL-17A+TNFα) *in vitro*. **(C)** Semi-quantification of the bands for LC3A/B, Atg5, and Beclin1 and SQSTM1. Bar is means ± SD of three separate experiments. *P <0.05, **P < 0.01, and ***P < 0.001 vs. control for activated (IL-17A+TNFα) only and activated with keratinocytes treated with fisetin or rapamycin. When contrasting the treatment groups for fisetin and rapamycin, the # is used. * is used when we compared with the activated group. ns, not significant.

Based on our RNA-Seq data analysis that identified the upregulation of multiple autophagic related genes in the fisetin-treated and TNF-α/IL-17A-stimulated keratinocytes, we next examined the protein expression levels of the core autophagosomal-lysosomal autophagy marker light chain 3 (LC3). By immunofluorescent staining analysis, the effects of fisetin (15 µM) or rapamycin (100 nM) pretreatment of keratinocytes prior to stimulation with TNF-α/IL-17A for 36 h on autophagy-related marker, LC3A/B (green) and cytoskeleton marker, Actin (Red) was examined. TNF-α plus IL-17A stimulation did not increase LC3A/B staining in the keratinocytes when compared to control (**Figure 3A**). By contrast, both fisetin and rapamycin pre-treatments prior to cytokine-activation resulted in increased LC3A/B punctate staining in the cells, which is consistent with the RNA-seq findings. ImageJ analysis of the fluorescence intensity revealed that fisetin considerably stimulated autophagy, equivalent to the known autophagic inducing effect of rapamycin (**Figure 3C**, left). This findings was further validated by Western blot analysis of whole cell lysates, which revealed elevated protein expression levels of Atg5, Beclin-1 and SQSTM1 in fisetin, rapamycin or their combination pretreated and activated keratinocytes (**Figures 3B, C**).

### Fisetin directly interacts, binds and inhibits IL-17A production by activated CD4^+^ T lymphocytes when cultured alone and when co-cultured with keratinocytes *in vitro*

**Figure 4:**
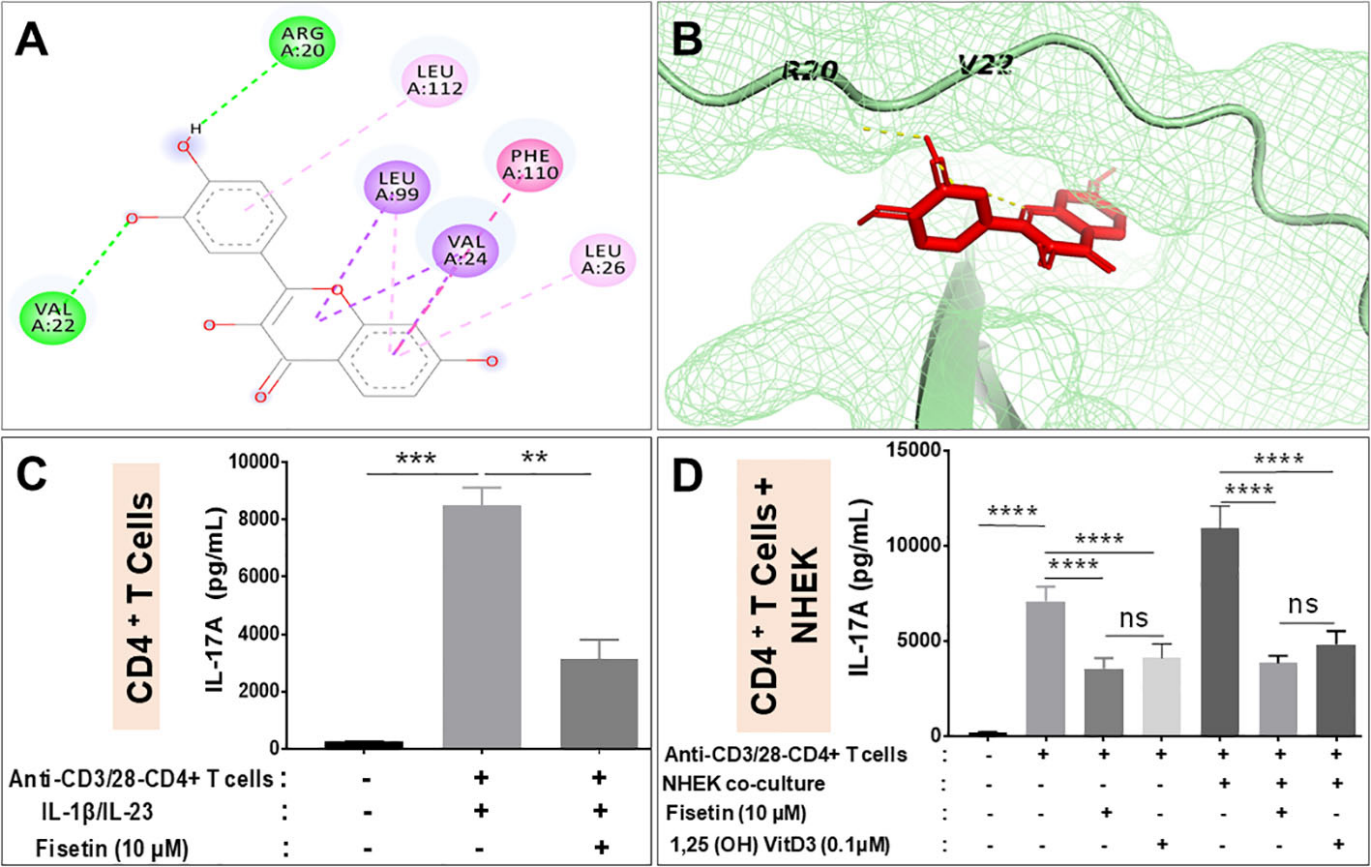
Fisetin reduces IL-17A production by activated CD4^+^ T lymphocyte alone and when co-cultured with keratinocytes *in vitro.* **(A)** Binding affinity of fisetin to IL-17A, predicted using Autodock vina and interaction of fisetin in binding pocket of IL-17A shown in stick and mesh model. **(B)** This interaction involves hydrophilic and hydrophobic interactions with different residues shown in 2D layout. ELISA assay for the levels of IL-17A secretion by anti-CD3/anti-CD28 activated purified human CD4+ T lymphocytes under different conditions: **(C)** anti-CD3/anti-CD28 activated CD4+ T lymphocytes cultured alone and then primed with IL-1β and IL-23 to enhance IL-17A production in the presence or absence of fisetin. **(D)** anti-CD3/anti-CD28 activated CD4+ T lymphocytes co-cultured with NHEKs in the presence or absence of fisetin (10 µM) or Vit-D3 (0.1 µM). Cells were cultured for 48 h in the indicated conditions and secreted IL-17A in the condition culture media was assayed by ELISA. Bar represents Means ± SE resulted from at least three separate experiments and paired *t* test was used to compare values between treatments. **P <0.01 and ***P <0.001 (–). indicates non-activated or treated cells. ns, not significant.

We determined whether fisetin physically interact and/or binds IL-17A by conducting intensive *in silico* molecular docking analysis using the autodock vina program. This analysis predicted physical interaction and demonstrated strong binding of fisetin with IL-17A with a binding energy of - 7.1 Kcal/mol with a good RMSD of 1.928. Fisetin also binds IL-17RA with a binding energy of -6.9 Kcal/mol with a higher RMSD of 2.378, confirming a higher binding affinity with IL-17A as the preferred site than its receptor. The hydroxyl groups of fisetin forms hydrogen bond interaction with Arg20, val22, while val24, Leu26, 99 and 112 and Phe110 of IL-17A forms hydrophobic interaction (**Figures 4A, B**).

We next examined the effectiveness of fisetin in inhibiting cytokine production, particularly IL-17A by human CD4^+^ T cells under Th17-polarizing (anti-CD3/anti-CD28) conditions or by co-culturing activated CD4^+^ T cells with keratinocytes in the presence of fisetin or Vitamin D_3_. Activation of CD4^+^ T cells by anti‐CD3/anti-CD28 in the presence of IL‐1β and IL‐23 resulted in significant IL-17A production (**Figure 4C**). However, pretreatment of CD4^+^ T cells with fisetin (10 µM) before the addition of IL‐1β and IL‐23 resulted in significant inhibition of IL-17A release by anti‐CD3/anti-CD28 activation (**Figure 4C**). The outcome of the interaction of skin keratinocytes and CD4^+^ T cells with regards to IL-17A production was examined the by co-culturing keratinocytes in the presence of activated CD4^+^ T cells with or without test compounds. Therefore, after CD4^+^ T cells acquired memory phenotype as described above, T cells were treated or not with fisetin or Vitamin D_3_, and cell free supernatant analyzed by ELISA. As observed above, anti‐CD3/anti-CD28 activation of CD4+ T cells resulted in significant release of IL-17A. However, a more significantly robust and higher IL-17A production was observed when anti‐CD3/anti-CD28 activated CD4+ T cells were co-cultured with keratinocytes (NHEKs) than when CD4+ T cells were cultured alone (**Figure 4D**). More interestingly, pretreatment with fisetin (10 μM) was observed to significantly decrease the amount of IL-17A production induced upon co-culture of activated CD4+ T cells with NHEKs. It was observed that these decreases in inflammatory responses were comparable to values obtained after treatment withVit-D_3_ (0.01 μM), a known positive control compound whose analogs are current mainstay for treating psoriasis (**Figure 4D**), which is in line with a previous report (42). Therefore, these data indicate that fisetin strongly inhibits critically important pro-inflammatory cytokines including IL-17A release by CD4+T cells when cultured alone or in co-culture with epidermal keratinocytes.

### Fisetin significantly improves IMQ-induced psoriatic skin lesions and reduces epidermal hyperplasia in mice

**Figure 5:**
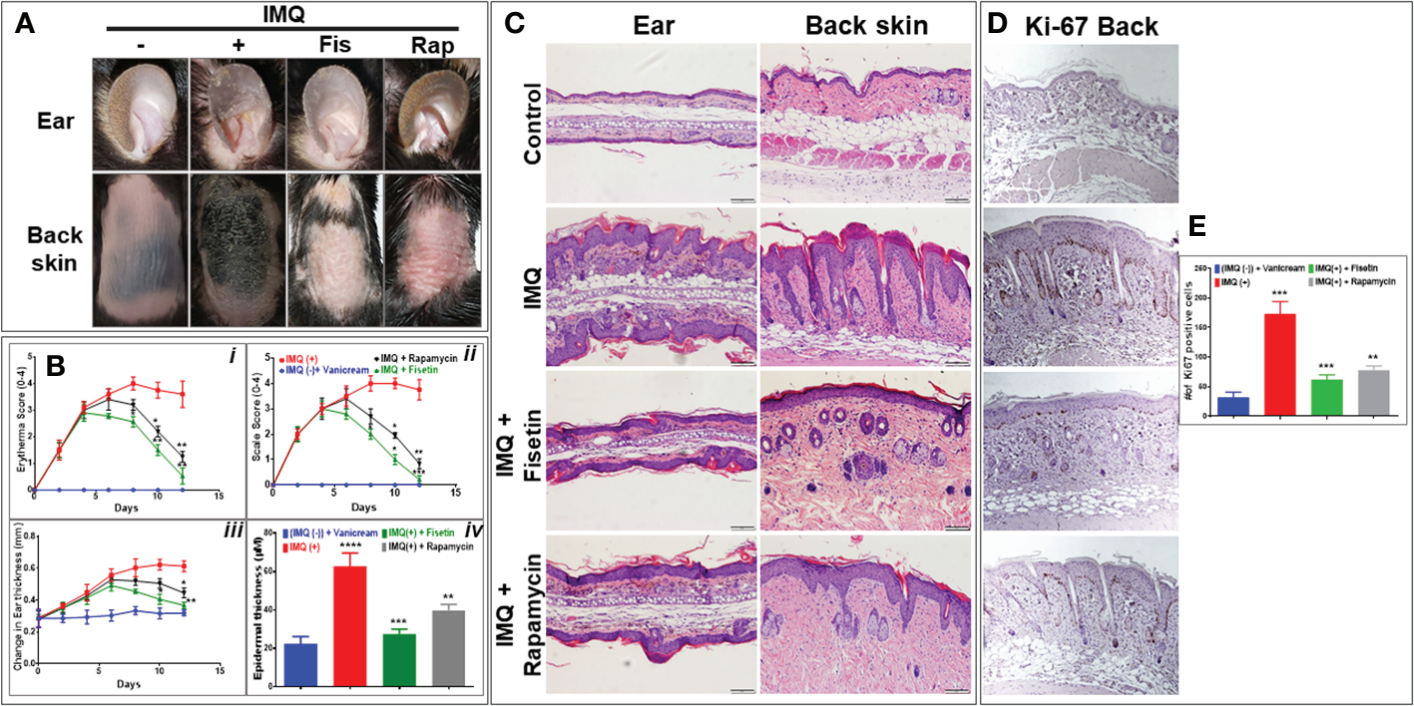
Fisetin improves clinical scores, alleviates epidermal hyperplasia, and reduces Ki67 in epidermis of IMQ-induced skin lesion in C57BLC6 mice. **(A)** Representative preclinical photographs of ears, and back skin of IMQ-treated C57BLC6 mice treated topically once daily for 10 days with fisetin (50 mg/kg body weight) or rapamycin (25 mg/kg body weight) compared with vehicle (vanicream) control mice. Psoriasis Area and Severity Index (PASI) was used for scoring. Scale bar = 100 µm. Schematic overview of Timeline of IMQ studies. (**B**: *i-iii*) Four blinded evaluators scored ears, and skin for erythema, scaling, and ear swelling (thickness). Scoring ranged from 0 to 4, increments of 1, with 0 indicating normal phenotype and 4 indicating a severe psoriasis-like phenotype. **(C)** Histological H&E-stained ear and skin sections from mice treated with vanicream control (i.e., no Aldara reference), IMQ, fisetin+IMQ, or rapamycin+IMQ. (B; *iv*) Microscopic quantification of average interfollicular epidermal thickness of vanicream control, IMQ, fisetin_IMQ, or rapamycin+IMQ treated mouse skin sections. **(D)** Immunohistochemical staining of skin sections for Ki67. **(E)** Quantified numbers of Ki67-positive cells in basal and suprabasal epidermis from mice with different treatments. Scale bar = 100 µm. The epidermal thickness scoring and Ki67-positive cell average count was calculated from each group (n=6). Four areas per section of a sample were sampled and analyzed. Means ± SE are shown, and paired *t* test was used to compare values between different treatments. *P <0.05, **P <0.01 and ***P <0.001. (-) indicates control group.

We next established the *in vivo* relevance of our *in vitro* findings by evaluating the effects of topical fisetin and rapamycin application in the IMQ-induced mouse model of psoriasis. Topical doses of 50 mg/kg of fisetin, 20 mg/kg of rapamycin, or vehicle vanicream control were administered daily to C57B6J mice for a maximum of 12 days beginning at 7-8 weeks old (n = 6, **Figure S3A**). In order to clarify the difficult lesions observed in C57BL6 mice skin lesion when IMQ was applied, we also employed a simple BALB/c mice model to show clear pictures of lesions (**Figure S3B**). The clinical Psoriasis Area and Severity Index (PASI) score was recorded by four blinded researchers on a scale of 0-4, with 4 representing a severe phenotype. Ears and back skin were evaluated for erythema, scaling, and bifold skin thicknesses. Mice treated with fisetin + IMQ or rapamycin + IMQ presented a significantly reduced severe skin/ear phenotype and evaluation scores compared to mice treated with IMQ alone (**Figures 5A, B**). Rapamycin lessened the skin/ear inflammation, scaling, and thickening induced by IMQ in mice, but fisetin showed even greater effect (**Figures 5A, B**). Both fisetin and rapamycin treatments inhibited daily increases in IMQ-induced inflammation (erytherma), scaling, and ear thickening (**Figure 5B** top panel, and lower left panel). For clearly appearing skin inflammation lesion induced by IMQ see **Figure S3B**. Morphometric analysis of H & E-stained sections showed that topical application of either fisetin or rapamycin significantly reduced skin/ear epidermal thickness with fisetin treatment leading to a greater improvement (**Figure 5B**; lower right panel). Micrographs of H & E-stained sections and epidermal thickness morphometric analyses revealed that compared to the vanicream-treated controls (**Figure 5C**; top panels), the IMQ-treated ear and back skin showed thicker epidermis with hyperparakeratosis, increased epidermal rete ridges, and increased immune cell infiltration and Munro microabscesses (**Figure 5C**; second panels). In contrast to the ear and back skin treated with IMQ, those treated with fisetin + IMQ showed dramatically decreased epidermal thickness, immune cell infiltration, and epidermal rete ridges (**Figure 5C**; third panels). Rapamycin treatment equally alleviated IMQ-induced lesions (increased epidermal thickness and rete ridge area, and hyperplasia) (**Figure 5C**; lower panels). Next, we evaluated the proliferative activity of the epidermis by assessing the expression of the cell cycle marker Ki67 in cells proximal to the basement membrane zone. We observed that IMQ-induced heightened psoriasiform hyperproliferation in the skin compared to vehicle control and that both fisetin and rapamycin treatment significantly reduced IMQ-induced epidermal hyperproliferation in the skin tissue **Figures 5D, E** and (data not shown).

### Fisetin treatment modulates the activation of mTOR/Akt/STAT3 signaling and suppresses inflammation, immune mediators release and angiogenesis in mice

**Figure 6:**
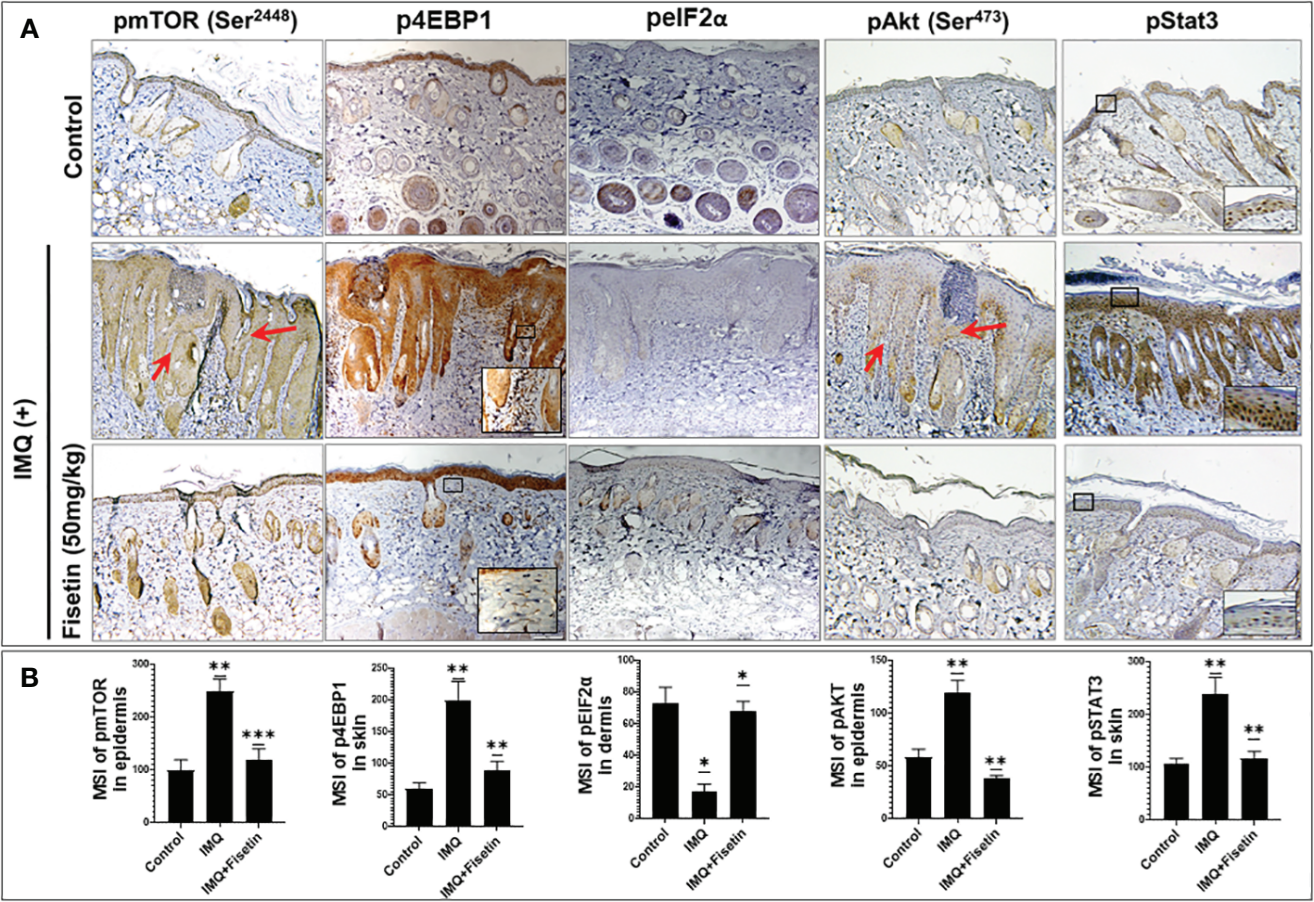
Fisetin inhibits the phosphorylation of mTOR, 4EBP1, Akt and Stat3 and increased the phosphorylation of eIF2α in IMQ-treated skin of mice. **(A)** Immunohistochemical staining of p-mTOR (Ser^2448^), p-4EBP1(Thr^37/46^), p-eIF2α (Ser^51^), p-Akt (Ser^473^) and p-Stat3 (Tyr^705^), in fisetin- or vehicle-treated in IMQ-induced mouse dorsal skin sections. Scale bar = 100 µm. **(B)** Quantified intensity of p-mTOR, p-4EBP1 and p-eIF2α, p-Akt and p-Stat3 per 20× microscopic field view. The mean intensities and quantifications are shown in bar graphs as mean ± SE, and paired *t* test was used to compare values between the different treatments. These were calculated from each group (n=6). *P <0.05; **P <0.01 and ***P <0.001. (-) indicates control group.

To better understand the role of fisetin on the epidermal, fisetin was applied topically on IMQ-treated mouse skin for 9 consecutive days and further examined its effectiveness on the Akt, mTOR, and its effector p70S6K pathways *in vivo* in mice. Consistent with previous findings (15), immunohistochemical staining showed that compared to vehicle treatment, IMQ-treatment significantly increased the phosphorylation of mTOR, eukaryotic translation initiation factor 4E binding protein 1 (4EBP1), and Akt in mouse skin. Next we found that fisetin treatment resulted in inhibition of mTOR and induction of autophagy, leading to preclinical alleviation of IMQ-induced psoriasis-like skin inflammation, skin thickness and scaling (**Figure 6**). Increased phosphorylation of eukaryotic translation initiation factor 2A (eIF2α) known to inhibit cap-dependent translation (43), was observed to be reduced upon IMQ treatment (**Figure 6**), suggesting the activation of cap-dependent translation, which is in line with increased cell proliferation in the skin (see **Figure 5D**). Fisetin treatment also rescued IMQ-induced reduction of phosphorylated eIF2α, particularly in the hair follicles, which is consistent with a recent study showing that application of Albendazole to IMQ-treated lesions resulted in increased expression levels of phosphorylated eIF2α (44).

Further, immunohistochemical staining revealed that fisetin treatment suppressed IMQ-induced phosphorylation of signal transducer and activator of transcription 3 (STAT3) in mouse skin to the control level (**Figure 6**). STAT3 signaling regulates epidermal keratinocyte and immune cell responses, and hyperactivation of STAT3 signaling plays a critical role in the pathogenesis of T cell related diseases such as psoriasis (45–48). For this, we next examined the impact of fisetin on IL-22 production by myeloid lineage cells (dermal mast cells, Langerhans cells), and CD4+ effector T cells as well as on the expression of iNOS and angiogenesis (neovascularization).

Immunohistochemical staining showed that fisetin treatment suppressed IMQ-induced expression of IL-22 and iNOS in the skin to the control level (**Supplementary Figure S4** online). The presence of endothelial cells in a tissue, identified using the marker cluster of differentiation 31 (CD31), largely enhances angiogenesis. A vertically arranged cluster of CD31-positive cells in the dermis and altered epidermal appearance are both favorable indicators of psoriasis (36, 49, 50). We examined the effect of fisetin treatment on the psoriatic skin vasculature (angiogenesis). By immunohistochemical staining. A significant increase in the dermal CD31-positive cell labeling was observed in IMQ-treated skin compared to the vehicle-treated skin; fisetin treatment significantly mitigated IMQ-induced expression of dermal CD31^+^ positive labeling (**Supplementary Figures S4A, B** online).

### Fisetin treatment enhances differentiation, increases filaggrin and loricrin protein expression and inhibits non-T and- T-lymphocyte infiltration in IMQ-treated lesions in mice

**Figure 7:**
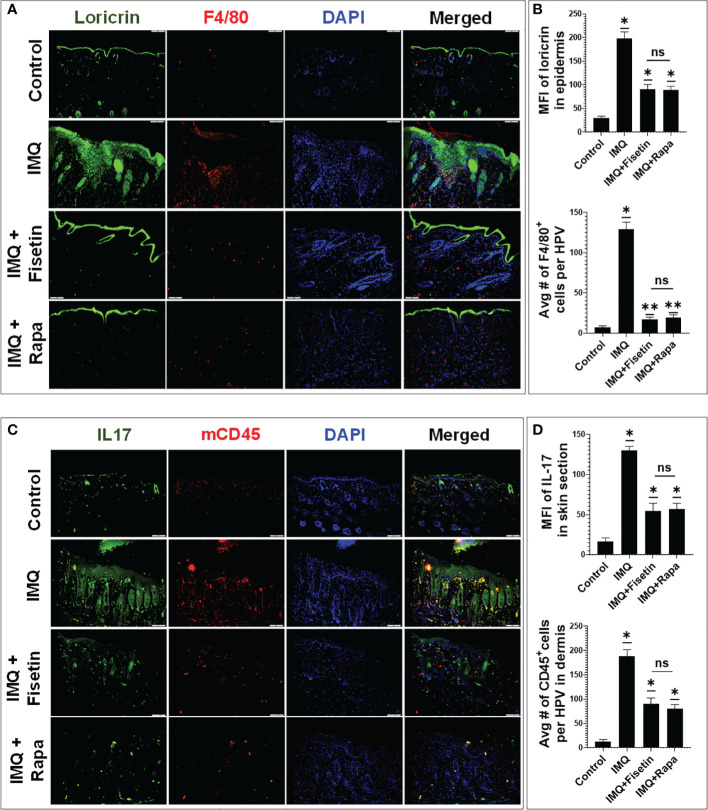
Fisetin normalizes and enhances the expression of differentiation marker loricrin, and reduces the expression of IL-17 and the infiltration of F4/80+ and pan-CD45+ immune cells in IMQ mice skin lesions. **(A)** Immunofluorescence staining of loricrin (green) and macrophage cell activation marker F4/80 (red) in vehicle-, IMQ-, and fisetin+IMQ or and rapamycin+IMQ-treated mouse skin. **(B)** Quantification of the fluorescent intensity of loricrin and F4/80+ cells per 20× field view. **(C)** Immunofluorescence staining of IL-17 (green) and pan CD45+ T lymphocytes (red) in vehicle-, IMQ, fisetin+IMQ-treated, and rapamycin+IMQ-treated mouse skin. DNA in nuclei was counterstained with DAPI (blue). The merged image shows colocalization of IL-17 with some CD45+ cells, which delineates IL-17^+^CD45^+^ T lymphocytes especially in IMQ-treated mice. **(D)** Quantification of the fluorescent intensity of IL-17 (green) and pan CD45+ T lymphocytes (red) per 20× field view. Shown in bar graph are mean± SD, and paired *t* test was used to compare values between different treatments, average reading from each group (n=6). *P <0.05 and **P <0.01. (-) indicates control group and ns indicates non-significant.

As aberrant differentiation associated with decreased expression of major structural cornification proteins, loricin and filaggrin are hallmarks of psoriatic epidermis (51 – 51),, we determined the effect of fisetin treatment on their expression levels and distribution in IMQ-induced skin lesions by immunofluorescence microscopy. While IMQ treatment resulted in decreased, and abnormally widespread expression of loricrin (**Figure 7**; green) and filaggrin (**Supplementary Figure S5**; green) in the basal proliferative skin layers, fisetin or rapamycin treatment blocked these effects. Fisetin significantly normalized IMQ-related responses and induced the protein expressions of loricrin (**Figure 7**; green) and filaggrin (**Supplementary Figure S5**; green) confined in the differentiating epidermal layers (**Figures 7**, **Figure S5** online), and these effects were comparable to that of rapamycin.

The Th1 (TNF-α) and Th17 (IL-17A and IL-22)-related cytokines are important proinflammatory cytokines, involved in maintenance of chronic inflammatory response and epithelial tissues remodeling in psoriasis pathogenesis (52–56). To assess the effect of fisetin as well as rapamycin on infiltrating T and non-T immune cells, we immunoassayed skin sections and analyzed the expression of markers of these cells by immunofluorescence microscopy. We observed that exposure to IMQ significantly increased the percentage of F4/80^+^ macrophages in the dermal skin compared to vanicream vehicle treatment, which was significantly suppressed by fisetin or rapamycin treatment at comparable levels (**Figure 7**; Red). Next, we assessed the effects of fisetin on T lymphocyte infiltration in skin lesions by performing immunofluorescence staining for CD4^+^ and pan-T lymphocytes markers (mcCD45). Fisetin treatment significantly attenuated the IMQ-induced increases in positively stained mcCD45^+^ (**Figure 7C**; Red) and CD4^+^ (**Supplementary Figure S5**; Red) T lymphocytes in the back-skin lesion in mice and at values comparable to that of rapamycin. Elevated pro-inflammatory cytokines in psoriasis cause neutrophil accumulation and epidermal alterations resulting in a neutrophilic microabscess (57–60). Also, myeloid cells are important producers of TNF-α, iNOS, and IL-23, which promote the production of IL-17A by CD4+ T cells (61, 62), and contribute to cardiovascular comorbidities related to psoriasis (63). Thus, we examined the impact of fisetin as well as rapamycin on IL-17 secretion and expression by Th17 cells during IMQ-induced inflammation in mice. A considerably increased protein expression levels of IL-17 (**Figures 7C, D**) and IL-1β (**Supplementary Figure S6**; green online) was observed in IMQ-treated histologic skin sections. Nonetheless, fisetin treatment of IMQ-lesions resulted in significantly reduced expression of IL-17 (**Figure 7C** (green) and IL-1β (**Supplementary Figure S6** (green)) compared to IMQ-treated alone skin sections to levels similar to that of rapamycin. Moreover, we observed co-localization (yellow) of IL-17-positive T lymphocytes (IL-17^+^CD45^+^ cells) in the dermal component of IMQ-alone treated skin sections (**Figures 7C, D**; yellow). Therefore, histologically, there was decrease in epidermal hyperplasia and skin infiltration of CD4^+^, CD45^+^ and F4/80^+^ cells after fisetin or rapamycin treatment, suggesting reduction of non-T and T-cell activation.

### Fisetin treatment induces autophagy and reduces serum LDL to HDL ratio in IMQ-induced psoriatic lesions in mice

**Figure 8:**
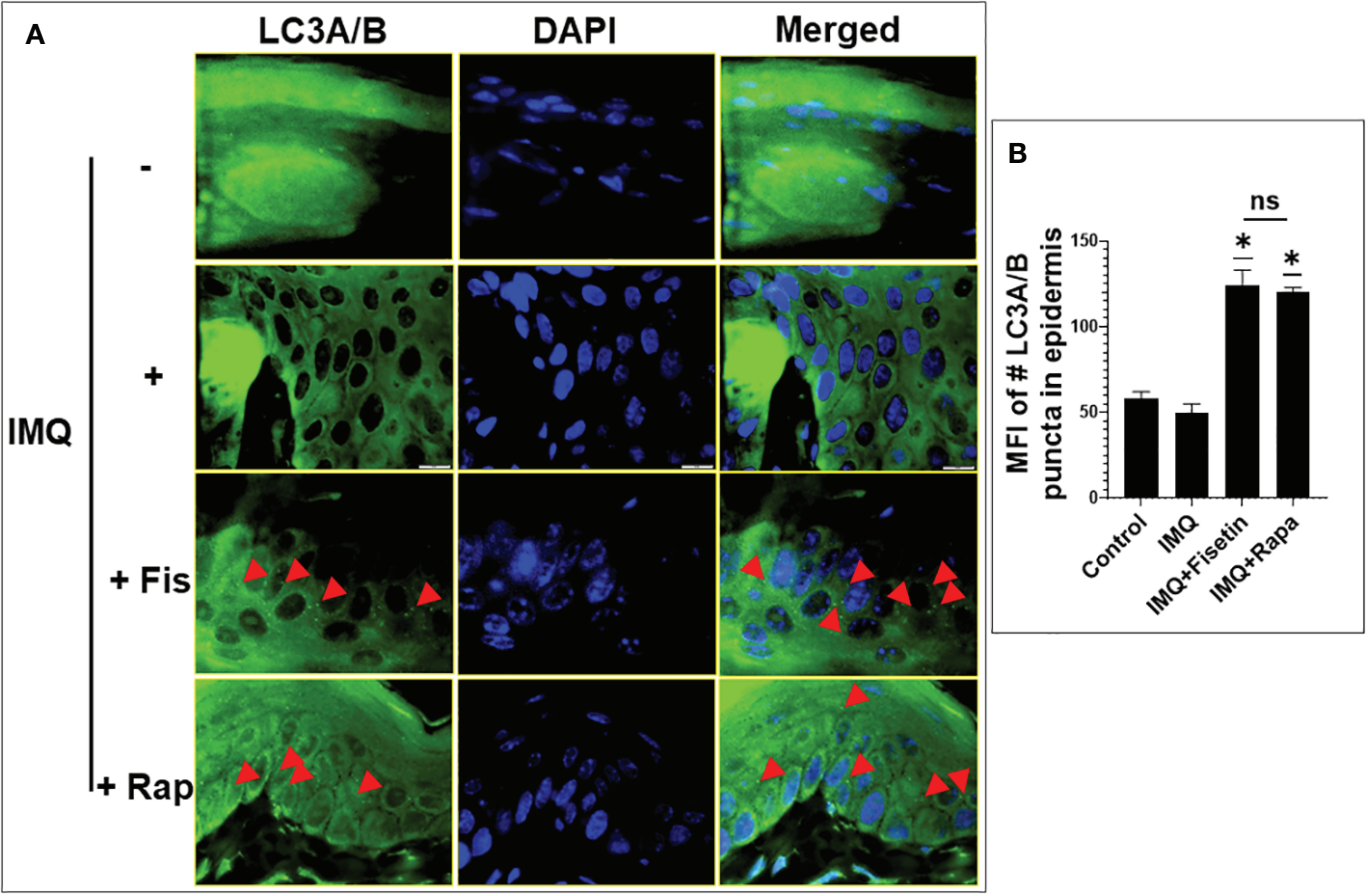
Fisetin induces the expression of autophagy marker LC3A/B in IMQ-induced skin lesion in mice. **(A)** LC3A/B (green) immunofluorescence staining and DAPI (blue) staining (for nucleus) in the skin tissue sections of vehicle-, IMQ-, fisetin+IMQ- and rapamycin+IMQ-treated mice. Red arrow heads in the fisetin+IMQ or rapamycin+IMQ treatment indicates green punctate staining of LC3A/B, which is absent in the control or IMQ treatment. Scale bar = 10 µm. **(B)** Quantification of fluorescent intensity of LC3A/B-positive puntata per 60x field view. Shown in bar graph are mean ± SD, and paired *t* test was used to compare values between different treatments, number of mice per group n=6. *P <0.05 and ns is non-significant.

Suppression of autophagy in keratinocytes amplify the inflammatory response in psoriasis (31, 64, 65). Since our *in vitro* transcriptomic and protein expression data indicated that fisetin induced autophagy in TNF-α/IL-17A-induced primary keratinocytes (**Figures 1**, **3**), we examined the effect of fisetin or rapamycin on autophagy induction *in vivo* in IMQ-treat mice skin lesions. Consistent with a previous report (64), immunofluorescence staining for autophagy marker LC3A/B indicated that IMQ-treatment alone did not induce autophagy in epidermal keratinocytes in mice skin sections as evidenced by the absent of green punctate staining (**Figure 8**, green) when counterstained with DAPI (blue). However, the numerous punctate immunofluorescently stained LC3A/B (red arrow head) in mice skin sections showed that topical application of fisetin significantly increased autophagy in IMQ-treated mice skin lesions to values similar to rapamycin (**Figure 8**).

### Fisetin treatment suppresses the expression of immune mediators *in vivo* in mice skin lesions

Last, we assessed the differences in cytokines, chemokines and growth factors production in lesioned and non-lesioned mice skin lysates collected from different mice groups after topical treatment with compound (fisetin or rapamycin) or vehicle control vanicream using the Luminex-based ProCarta 36-Plex ELISA assay. Importantly, topical application of fisetin significantly decrease production of the different Th1 and Th17 pro-inflammatory cytokines and chemokines, including other mediators for neutrophil and monocyte recruitment to the site of inflammation in the IMQ-induced psoriasis-like mouse model (**Table 1**). Topical rapamycin treatment was equally observed to decrease and modulate several of these immune-mediators in skin lesions (see **Table S1** online). Immune mediators that were not significantly reduced or modulated are shown in supplementary data files (**Table S2**).

**Table 1:** List of immune mediators that are significantly modulated upon treatment of IMQ-induced mice skin lesions with fisetin or rapamycin. Each group of mice had 6 mice (n=6) and control group was n=5.

Cytokine/Chemokine	Sensitivity (pg/mL)	Standard Curve Range (pg/mL)	Control (n=5)	Imiquimod (n=6)	Imiquimod + Fisetin (n=6)	Imiquimod + Rapamycin (n=6)
Pro-inflammatory						
TNF alpha	0.39	3.7-15000	17.2±4.0	28.7±3.2**	5.5±1.0***	11.5±2.0**
IL-1 beta	0.14	1.2-5000	15.5±3.02	27.9±3.5**	7.4±2.2 ***	14.0±3.2 **
IL-1 alpha	0.32	3.5-14300	527.7±57.2	774.3±67.1**	357.6±47.7****	449.6±67.7***
IL-6	0.21	4.9-20000	18.0±0.7	33.3±3.6***	9.7±3.3***	16.3±5.7**
IL-18	9.95	36.6-150000	41.3±21.4	122.6±2.2***	22.29±2.3***	52.29±9.3**
Type-1						
IFN gamma	0.09	1.2-5000	6.6±2.2	11.4±1.6**	6.9±2.1**	8.4±1.4*
IL-12p70	0.21	2.4-10000	0.6±0.02	3.6±.025**	0.6±.018**	1.2±.018*
IP-10 (CXCL-10)	0.26	0.5-2000	41.6±14.6	105.04±9.4***	56.2±16.6***	78.4±19.2**
IL-27	0.34	2.4-10000	134.1±17.0	223.3±50.3***	84.1±29.4***	124.1±29.4**
Type-17						
IL-17A	0.08	1.2-5000	523.0±118	1006.5±75.3****	223.6±171.6****	478.3±211.5**
IL-23	2.21	12.2-50000	19.6±6.3	137.4±4.8****	23.9±3.6****	84.1±17.4**
Type-22						
IL-22	0.24	12.2-50000	138.3±15.8	737.2±49.1****	119.0±9.5****	325.0±7.5***
Type-4						
IL-13	0.16	2.4-10000	3.6±0.9	9.5±1.2**	5.8±0.8*	6.9±1.1*
IL-4	0.03	1.2-5000	6.5±1.3	53.04±0.4***	3.9±1.2****	19.9±2.2**
IL-5	0.32	2.4-10000	17.8±3.1	40.4±3.2***	9.7±4.1***	22.2±6.7**
Eotaxin (CCL-11)	0.01	0.5-2000	772.9±167.1	2235.1±592.8****	955.6±214.4****	1213±253.8***
Regulatory						
IL-10	0.69	2.4-10000	335.0±59	586.1±76.7***	766.2±52.75****	696.2±82.75***
Neutrophil chemoattractant						
Gro alpha (CXCL-1)	0.05	1.7-7000	91.9±10.0	123.6±23.4**	26.6±24.1****	56.9±30.1**
MIP-2 (CXCL-2)	0.37	0.7-3000	2609.7±592.7	4434.6±656.4****	1552.8±103.5****	2231.3±103.5**
Monocyte/hematopoetic cell chemoattractant						
MCP-1 (CCL-2)	3.43	7.3-30000	32.3±13.2	63.9±9.1**	38.6±6.8**	45.9±11.2**
MCP-3 (CCL-7)	0.15	0.2-1000	452.5±22.3	605.5±41.5***	313.2±57.7****	401.5±11.3**
MIP-1alpha (CCL-3)	0.13	0.5-2000	448.2±90.7	693±53.1***	185.8±56.1****	297.2±41.3**
MIP-1beta (CCL-4)	1.16	1.2-5000	12.5±3.1	31.4±7.1**	5.0±1.0***	10.4±1.4**
Hematopoietic cell differentiation						
M-CSF	0.02	0.3-1100	127.8±19.1	218.7±24.5***	52.0±18.5****	82.7±16.2**
**Other**: LIF	028	2.4-9850	150±12.9	203.0±27.7**	30.7±4.6****	67.1±9.2***

Each group of mice had 6 mice (n=6).

*P <0.05; **P <0.01; ***P <0.001 and ****P<0.0001.

## Discussion

This study demonstrates that fisetin and rapamycin suppressed cytokine-induced keratinocyte associated inflammatory responses, including Akt/mTOR signaling, autophagy and the production of proinflammatory cytokine IL-17A by activated CD4^+^ T-lymphocytes. Furthermore, the *in vivo* relevance indicated that topically applied fisetin promoted epidermal keratinocyte autophagy and differentiation, and alleviated IMQ-induced psoriasiform lesion in mice. The immune mediators released by activated keratinocytes and immune cells, including T lymphocytes, play key roles in perpetuating/enhancing hyperproliferation, aberrant differentiation, resistance to apoptosis and hyperactivation of the PI3K/Akt/mTOR signaling in lesioned psoriatic epidermis (66–70). However, the perturbed pro-inflammatory epidermis creates a positive feedback loop that further activates keratinocytes to produce more cytokines, which further exacerbate the inflammatory response (69–72). Therefore, suppression of keratinocyte hyperproliferation, T-lymphocyte activation as well as the release of their proinflammatory mediators while inhibiting the activation of the central mTOR have proven to be useful clinical anti-psoriasis strategies (70, 73–77).

Many current psoriasis treatments, including dithranol, methotrexate, vitamin D3 analogs or PUVA phototherapy modulate keratinocyte differentiation (78, 79). In spite of recent research advances, there remains a dire need for enhanced psoriasis therapies (80, 81). Current systemic biologic therapies targeting cytokines (Th1, Th17, and Th23) and their receptors are most beneficial (82). Natural products such as flavonoids have been investigated for treating psoriasis, targeting apoptosis as a strategy against keratinocyte hyperplasia (83). In pursuit of safe and effective anti-psoriasis agents, a novel non-toxic naturally occurring dietary polyphenolic small molecule, fisetin (3’,4’,7-trihydroxyflavonol) has been identified. Fisetin is abundantly found in pigmented fruits and vegetables, has a lower cost of production than traditional synthetic drugs (84, 85), and is shown to have beneficial effects in regulating metabolism and in treating several ailments including cancer and inflammation (31, 34, 86). Moreover, since fisetin has poor oral bioavailability and is primarily metabolized by intestinal microbiota, we herein employed topical fisetin delivery.

The IMQ-induced mouse model, a known murine psoriasiform dermatitis model used in several hundred of studies, was used to provide preclinical proof-of-concept data that extend our *in silico* and *in vitro* findings. IMQ is an agonist of the Toll-like receptor 7/8 ligand, and its application histologically closely resembles human psoriasis (87). This mouse model of psoriasis displays/shows a skin phenotype closely resembling human psoriasis including marked psoriasiform hyperplasia, scaling, erythema, and thicknesses a characteristic contribution of both non-T and T immune cell infiltrations motivated by the IL-17/IL-23 axis, and thus it is ideal for studying fisetin therapy (87–92). Consequently, we hypothesized that dual inhibition of immune cells and targeting the mTOR/autophagy arm with a single natural product would alleviate the psoriasis-like phenotype of IMQ-induced mice. The IMQ model displays sex and mouse strain disparities, and female B6 background mice have transcriptional signatures that closely resemble psoriasis (89). Additionally, as the ratio of males (2.8%) to female (3.8%) human psoriasis patients are very close, we employed female B6 mice to avoid misinterpretation of disease characteristics. However, the IMQ model exhibits numerous limitations, including inability to analyze them for long-term (93). Here, we found that fisetin treatment diminished IMQ-induced upregulation of TNF-α, IL-22 and IL-17A in mouse skin lesions, while there were no changes in IL-9. Activation of transcription *via* the STAT3 pathway appears to be the primary mechanism of IL-22 signal transduction by which IL-22 promotes IL-23-induced acanthosis and cutaneous inflammation in psoriasis through the activation of STAT3 (53, 94, 95). Also, dendritic cells produce IL-23 and IL-20, which can activate and differentiate T cells and keratinocytes, to express high levels of TNFα and innate immunity-related inducible nitric oxide synthase (iNOS) (13, 96–98). In this study, there was also a marked decrease in psoriasis-associated STAT3 activation in epidermal cells of IMQ-induced C57BJ6 mice after fisetin treatment, suggesting that fisetin disrupts the positive immune to skin trafficking feedback that drives psoriasiform dermatitis. Complete resolution of disease features was not achieved by either fisetin or rapamycin though much better results were seen in the fisetin-treated group. This could suggest contributions by mTOR-independent and other pleotropic signaling pathways.

Activation of the PI3K/Akt/mTOR and associated signaling cascade has been linked to psoriasis pathophysiology such that released proinflammatory cytokines aberrantly induce mTOR activation that subsequently promote psoriatic lesions and epidermal keratinocyte hyperplasia (67, 68, 99–103). Also, this pathway activation and inhibition of autophagy have been reported to modulate cholesterol levels in IL-17A mediated inflammatory responses in psoriasis (104). Therefore, pharmacological suppression of overexpressed PI3K/Akt/mTOR cascade is potentially a promising anti-psoriatic strategy (76, 105). In this study, we found that fisetin directly modulates the transcription of the components of the mTORC1 (Raptor), and mTORC2 (Rictor) as well as Deptor, and other effector components of the mTOR signaling pathway, which are overexpressed in psoriasis. Further, fisetin treatment decreased p-Akt, p-mTOR and p-p70S6K1 protein levels, an indication that fisetin-induced amelioration of inflammatory responses *in vitro* and *in vivo* is related to the inhibition of the mTOR central signaling. Fisetin treatment was also shown to decrease the expression of Akt2 mRNA and DEFB4A, a key contributor to the pathophysiology of psoriasis (106). Rapamycin, an mTORC1 inhibitor with known immunosuppressive and antiproliferative properties (107), has been reported to have systemic beneficial effects in patients (77, 108–110), and has been topically shown to ameliorate IMQ-induced psoriatic skin lesions in mice (76, 111, 112).

Aberrant differentiation, increased proliferation, and resistance to apoptosis are hallmarks of psoriatic epidermis. The insoluble precursor protein loricrin, expressed in the late granular epidermal layers and a major component of the cornified envelope (113, 114), is downregulated and abnormally spread in skin lesions of psoriasis patients and in murine psoriatic epidermis (105, 115, 116). Additionally, filaggrin (51, 117), another major structural cornification protein is mutated in some psoriasis patients, and the skin lesions in the majority of psoriasis patients and murine models exhibit reduced and abnormal diffused filaggrin protein expression (115, 118). Current study indicates that the IMQ-induced decreased and spreading of loricrin and filaggrin protein expression to the entire epidermis, was reversed to uphold an enhanced expression of these differentiation markers upon fisetin and rapamycin administration.

Cells survive in nutrient-limited environments by decomposing their cellular contents to maintain homeostasis, energy levels, and key components. Autophagy is an essential homeostatic process for intracellular quality control (degradation cargo pathway) in a healthy body where cytosolic constituents of cells including epidermal keratinocytes are targeted and transported to lysosome/vacuole for lysosomal degradation. In the cytoplasm, a cup-shaped membrane structure known as the phagophore is formed in response to autophagy activation. When autophagy occurs, double-membrane vesicles known as autophagosomes are formed, and these vesicles ingest organelles and cytoplasmic debris and transport them to lysosomes for destruction. When the phagophore has grown sufficiently to accommodate its substrates, it shuts and seals to form a double membrane structure known as an autophagosome. Over 31 autophagy-related genes (ATG) products has been identified to regulate autophagy (39). In mammals, the autophagic initiation complex is composed of ULK1/2, ATG13, ATG101, the conjugation system of the autophagosome formation needs LC3 and ATG12. mTORC1 inhibits the activity of the ULK kinase complex, which is constitutively made up of ULK1 or ULK2, ATG13, FIP200, and ATG101, by phosphorylating ULK1 and ATG13 in nutrient-rich circumstances. Depriving the body of nutrients causes mTORC1 to be inhibited, which in turn causes ULK1 and ATG13 to be dephosphorylated. Through an unidentified mechanism, active ULK1 kinase complex connects with recycling endosomes and exits in vesicles that also include ATG9A and/or the ATG12-ATG5-ATG16L1 complex (119–121). Aberrant autophagy has been reported in both atopic dermatitis and psoriasis patients and TNFα treated KCs (122) and associated with accrual of p62/SQSTM1, which is destroyed during autophagic process, an indication that the processing of the cargo is hampered already even during the enhancement of the onset of autophagosome formation (122). Studies have shown that inhibition of autophagy is associated with psoriasis pathogenesis (104, 111, 112).

Our data revealed increased transcriptional activity and protein expression levels of autophagy-related targets upon fisetin treatment *in vitro* and *in vivo* including stronger expression of critical autophagosome development markers LC3A and LC3B. We suggest that fisetin induces autophagy in two ways: First, by increasing the expression of ULK1/2 genes, which, in turn, facilitates the production and direct maturation of autophagosomes, or secondly, by inhibiting the activation of Akt and mTORC1, which in turn, utilizes ULK proteins to assist in the development of autolysosomes and pre-autophagosomes. In keratinocytes that have been exposed to TNFα (64), there is a disruption in the autophagosome’s ability to fuse with the lysosome; yet, in our study, fisetin induced the autophagosome-lysosome fusion to form autolysosome, prospectively. It is possible that fisetin’s ability to improve psoriasis-like dermatitis and reduce hyperkeratosis may be partially due to its ability to stimulate autophagy. Rapamycin also increased autophagy-related marker expression both in keratinocytes (and their combination synergistically induced SQSTM1 expression) and in IMQ-induced psoriatic mouse skin. Earlier studies proved that rapamycin thwarted 2,3,7,8-Tetrachlorodibenzo-p-dioxin (TCDD)-induced inhibition of autophagy by increasing autophagy markers in *in vitro*, *ex-vivo* and *in vivo* psoriatic models (111, 112). Our study is consistent with such findings and supports the role of rapamycin and fisetin as autophagy inducers employed and as singly agent or in combination.

## Conclusions

In summary, we show that fisetin decreases inflammatory responses induced by keratinocytes and CD4+ T lymphocytes, suppresses the Akt/mTOR signaling pathway, promotes autophagy and differentiation, and improves both the immune and epidermal phenotypes of IMQ-induced psoriasis-like skin lesions in mice. Together these data provide the pre-clinical evidence for further development of fisetin as a potential natural product- derived anti-psoriatic agent.

## Materials and methods

### Chemicals and reagents

Fisetin, rapamycin, and dimethyl sulfoxide (DMSO) were purchased from Sigma Chemical Co. (St. Louis, MO, USA). The antibodies for phospho-Akt (Ser473) (#4060), phospho-mTOR (Ser2448) (#5536), β-actin (#4970), PhosphoPlus^®^ p70 S6 Kinase (Thr389, Thr421/Ser424) Antibody Kit (#9430), LC3A/B (#12741), Atg5 (#12994), Beclin-1 (#3495), phospho-Raptor (Ser792) (#2083), phospho-AMPKα (Thr172) (#50081), AMPKα (#5831), GAPDH (#5174), phospho-eIF2α (Ser51) (#3597), phospho-4E-BP1 (Thr37/46) (#2855), anti-mouse IgG, HRP-linked antibody (#7076), and anti-rabbit IgG, HRP-linked antibody (#7074) were obtained from Cell Signaling Technology (Danvers, MA, USA). Recombinant human (rh) IL-6, IL-22, IL-17A, TNF-α, anti-CD3, anti-CD28, and biotinylated polyclonal goat anti-human IL-17A, anti-pan-mCD45 antibody (#MAB114) were from R&D Systems (Minneapolis, MN, USA). The recombinant human (rh) IL-1β was purchased from HumanZyme (Chicago, IL, USA), and rhIL23A, mouse anti-F4/80 were from eBioscience/Thermo Fisher (Waltham, MS, USA). The IL-1β (#sc-7884), IL-22 (#sc-14436), and IL-17(#sc-7927) antibodies were all obtained from Santa Cruz Biotechnology (Dallas, TX, USA). CD31 monoclonal antibody (2H8) (# MA3105), iNOS monoclonal antibody (4E5) (# MA5-17139), and IL-22 monoclonal antibody (C6) (#MA5-41620) were obtained from ThermoFisher Scientific (Waltham, MA, USA). Rabbit CD4(sp35) (#104R-14) was from Cell Marque (Rocklin, CA, USA), rabbit anti-loricrin (#ab24722) was from Abcam (Cambridge, UK), FITC conjugated filaggrin antibody (#PRB-417-100) was obtained from Covance (Dedham, MA, USA). Aldara cream 5% imiquimod was from Valeant Pharmaceuticals, North America LLC (Bridgewater, NJ, USA). Vanicream (#267401) was purchased from Morris & Dickson LLC (Shreveport, LA, USA). Total Cholesterol and Cholesteryl Ester Colorimetric/Fluorometric Assay Kit was obtained from Biovision Inc. (Milpitas, CA, USA). CELLnTEC progenitor cell culture medium was from ZenBio (Raleigh, NC, USA). Fetal bovine serum (FBS) was obtained from Life Technologies (Grand Island, NY, USA). Mouse Procarta™ 36-Plex kit was from eBioScience/Affymatrix (EPX060-15073; Santa Clara, CA, USA). Horseradish peroxidase (HRP) conjugated anti-mouse or anti-rabbit secondary antibody was obtained from PerkinElmer Amersham Life Science Inc. (Arlington Height, IL, USA), and bicinchroninic acid (BCA) protein assay kit was obtained from Pierce (Rockford, IL, USA). Chemiluminescence GE Healthcare Amersham ECL select™ Western detection blotting reagent (#RPN2235) were purchased from (GE Healthcare Life Sciences, New Jersey, USA). ChemiDoc MP Imaging System was from Bio-Rad (Hercules, CA, USA).

### Human subjects

The study protocols were approved by the University of Wisconsin-Madison Health Sciences Institutional Review Board. Informed written consent was obtained from subjects prior to participation. Studies were conducted according to the principles of the Declaration of Helsinki.

### Establishment and culture of normal primary keratinocytes and activation with TNF-α, IL-6, IL-17A, IL-22, and treatments with compounds

Primary normal human epidermal keratinocytes were established from neonatal foreskin (HEKn) and adult skin biopsies (HEKa) as described (123) or obtained from ThermoFisher Scientific. Cells were grown and maintained in EpiLife culture medium supplemented with 1% penicillin–streptomycin and 1% human keratinocyte growth factor (HKGS) (Life Technologies) for about 2 months (≈8 passages) as previously described (34). Stock solutions (10 mM) of fisetin and rapamycin were made in DMSO, which were further diluted in respective growth media for treatment of keratinocytes. Control cells were exposed to equal volumes of DMSO alone, corresponding to a maximum concentration of 0.1% (v/v) of DMSO which had no effect on cell viability. Cells were maintained at 37°C in a humidified atmosphere of 95% air and 5% CO2, and the growth media were changed every other day until cells reaching desired confluence (60-80%) prior to experimentation. To determine the effects of test compounds on mTOR activity in primary keratinocytes, near-confluent keratinocytes were pre-treated with or without fisetin (10-20 μM) for 3-8 h followed by co-stimulation with or without the combination of rhIL-6 and rhIL-22 (each at 20 ng/mL). Cells were harvested and lysates were prepared for assessment of mTOR activity as described below. For cytokine-induced keratinocyte inflammatory responses, near-confluent NHEKa cells were pre-treated with or without different doses of fisetin for 3 h, and further exposed to the medium containing fisetin and the combination of rhTNF-α (10 ng/ml) and IL-17A (20 ng/ml) for 24 h. Afterwards, cell culture media were collected and centrifuged, and the supernatants were stored at -80°C until ELISA. Meanwhile, the cells were harvested and processed for RNA preparation and RNA-Seq, or for Western blot analysis.

### Cell and RNA preparation, and sample quality control for RNA sequencing

HEKa cells were seeded in 6-well plates (5 × 105 cells in 1 mL of culture media per well), and allowed to adhere and proliferate. The cells were pre-treated with fisetin (15 μM) or rapamycin (100 nM) for 3 h, and then stimulated with or without rhTNF-α (10 ng/ml) plus rhIL-17A (20 ng/ml) for 24 h. Following a brief washing with PBS, Trizol reagent was added to rupture the cells, and the lysates were collected and stored at -80°C immediately. Total RNA was extracted from the Trizol lysates using the illumina RNA extraction protocol. Total RNA quantity and purity were assessed on a nanodrop device, and RNA integrity was examined using Agilent 2100 (AATI).

### Library construction, quality control and RNA-sequencing

Messenger RNA was purified from total RNA using poly-T oligo-attached magnetic beads. After fragmentation, the first strand cDNA was synthesized using random hexamer primers, followed by the second strand cDNA synthesis using either dUTP followed by end repair, A-tailing, adapter ligation, size selection, amplification, and purification. The directional library was ready for experiments after end repair, A-tailing, adapter ligation, size selection, USER enzyme digestion, amplification, and purification. The library was checked with Qubit and real-time PCR for quantification and with a bioanalyzer for size distribution detection. Quantified libraries were pooled and sequenced on an Illumina NovaSeq 6000 platform by Novogene (Sacramento, CA, USA) and 150 bp paired-end reads were generated.

### RNAseq data processing, differential gene expression, and enrichment analysis

Quality control raw data (raw reads) of fastq format were first processed through in-house perl scripts. RNAseq clean data (clean reads) were obtained by removing reads containing adapter, reads containing ploy-N and low-quality reads from raw data and the Q20, Q30 and GC content of the clean data were calculated. All the downstream analyses were based on the clean data with high quality and were analyzed as described in our published reports (124–126). Briefly, clean reads were aligned to the reference genome (hg38) and paired-end clean 2 reads mapping using Hisat2 v2.0.5 (127). Feature counts v1.5.0-p3 (128) was used to count the reads numbers mapped to each gene, and then FPKM value of each gene was calculated. Differential expression of genes with biological replicates was calculated using the *DESeq2* package (1.20.0) in the R software (http://www.r-project.org/), with Benjamini-Hochberg-adjusted *P*-values of 0.05 considered to be significant (124–126, 129). Absolute fold change of 2 was set as the threshold for significantly differential expression. Enrichment analysis of differentially expressed genes was performed using clusterProfiler R package (130), including Gene Ontology (GO), KEGG pathways (http://www.genome.jp/kegg/), Reactome database, Disease Ontology (DO), and DisGeNET pathways (126); and also (124–126). The corrected *P*-value less than 0.05 were considered significantly enriched.

### PPI analysis of differentially expressed genes

PPI analysis of differentially expressed genes was based on the STRING database, which contains known and predicted Protein-Protein Interactions.

### Western blotting

Western blotting was performed as described previously (86). Briefly, after washing with cold PBS, cells were incubated in ice-cold lysis buffer (50 mM Tris-HCl, 150 mM NaCl, 1 mM EGTA, 1 mM EDTA, 20 mM NaF, 100 mM Na_3_VO_4_, 0.5% NP-40, 1% Triton X-100, 1 mM phenylmethylsulfonyl fluoride (PMSF) (pH 7.4), with freshly added protease inhibitor cocktail (Protease Inhibitor Cocktail Set III, Calbiochem, La Jolla, CA) on ice for 30 min. The cells were scraped off, and cell lysates collected in microfuge tubes and passed through 22.5-gauge syringe needles to break up the cell aggregates. The lysates were cleared by centrifugation at 15,000 *g* for 30 min at 4°C. The protein concentration in the supernatants (whole cell lysates) were determined using a BCA protein assay kit (Pierce) according to the manufacturer’s protocol. Lysates were used or immediately aliquoted and stored at −80°C for further analysis. Then, 10-20 μg protein was resolved on 4-12% polyacrylamide gels and transferred to a nitrocellulose membrane. The blots were blocked in blocking buffer (7% fat-free dry milk or for phospho-protein detection with (5% BSA)/1% Tween 20; in 20 mM Tris-buffered saline (TBS), pH 7.6) for 45 min at room temperature. Membranes were incubated with appropriate monoclonal or polyclonal primary antibody in the blocking buffer for 2h at room temperature, or overnight at 4°C, followed by 3 × 5min washes, and incubated with appropriate secondary antibody conjugated with HRP. The membranes were exposed to Chemiluminescence substrate Amersham™ ECL Select™ Western Blotting Detection Reagent and the signals were documented and analyzed using the Bio-Rad ChemiDoc MP Imaging System as described (15, 131). Equal loading of protein was confirmed by stripping the immunoblotted membranes and re-probing for β‐actin, vinculin, or GAPDH. The results displayed are representative of at least three independent experiments.

### Preparation, culture and activation of human blood CD4+ T lymphocytes, co-culture with NHEK, and treatment with test compounds

The naïve CD4^+^ T lymphocytes were prepared from venipunctured peripheral blood mononuclear cells (PBMCs) obtained from healthy volunteer/donors with appropriate approval by University of Wisconsin-Madison Health Sciences Institutional Review Board as previously described (34, 132). Briefly, heparinized human blood sample was diluted 1:1 in HBSS, and overlaid above Percoll (1.090 g/ml). After centrifugation at 700× *g* for 20 min at room temperature the mononuclear cells were recovered from the plasma/Percoll interface. Naive CD4+ T cells were prepared by negative selection using the Miltenyi Biotec CD4+ T Cell Isolation Kit II. T cell subset purification was performed by positive selection with magnetic bead separation. Using this method, CD4+ T cell purity was typically >98% as determined by flow cytometry (132, 133).

We developed an *in vitro* model of CD4+ T lymphocytes activated with anti-CD3/anti-CD28 to acquire a memory type/effector phenotype (132). For activation, CD4+ T cells (2 × 106/ml) were cultured in 1 ml of complete medium (RPMI plus 10% FBS) with 1 μg/ml of plate-bound anti-CD3 plus 1 μg/ml of soluble anti-CD28 (clones 37407 and UCHT1, respectively; R&D Systems, Minneapolis, MN, USA) in a 24-well plate (Corning Costar, Lowell, MA, USA). In this polarization condition, after 48 h activation, CD4+ T cells had a memory/effector phenotype (CD45RO^+^ CD25^+^) as previously shown (15, 131). The cells were then cultured for an additional 24 h in complete medium in the absence of activator. After this stabilization time, CD4+ T cells were then treated with or without fisetin (10 µM) for 10 min before activation in a 48-well plate with anti-CD3/anti-CD28 (1 µg/ml) plus the cytokines, IL-1ß and IL-23A (2 ng/ml each) to shift the lymphocytes into a type-17 phenotype with amplified IL-17 production for 24 h (134). Subsequently, the cell free cultured supernatants were harvested for measurement of IL-17A by ELISA.

Keratinocyte hyperproliferation and increased infiltration of activated immune cells are characteristic hallmarks of the pathogenesis of psoriasis and other inflammatory skin diseases (60, 135–137). In addition, we examined the effect of keratinocytes interaction on IL-17 production, by co-culturing NHEK with activated CD4+ T cells that had been pre-activated for 48h with anti-CD3 plus anti-CD28. Briefly, after activated T cells acquired the memory phenotype as described above, they were then pre-incubated with or without fisetin or Vitamin-D3 prior to reactivation with anti-CD3/CD28. As earlier described (132), following 12h reactivation, the CD4+ T cells were then added, in the presence or absence of fisetin, to pre-treated sub-confluent monolayers of NHEK, followed by the co-culture for 36h prior to ELISA analysis of cell free supernatants. The T lymphocytes subset purification was performed by positive selection with magnetic bead separation (Miltenyi), and cytokine levels in the cell free supernatants were detected by ELISA as above.

### Molecular docking simulation

To study the potential interaction of fisetin with the IL-17A site, we employed the protein’s crystal structure (PDB: 4HSA). AutoDockTools was used to analyze the receptor and ligand, while AutoDock Vina 1.1.2 (http://autodock.scripps.edu) was used to molecularly dock fisetin with the receptor’s potential binding site. The pose with the lowest anticipated binding energy was utilized to identify the interacting residues of IL17A. For comprehensive interaction visualization, we utilized PyMOL and Discovery Studio. (version 3.5, Dassault Systèmes BIOVIA, San Diego) methodology has been described previously (138).

### ELISA for IL-17A levels

IL-17A levels in cell free cultured supernatant fluids from various conditions including activated or non-activated CD4^+^ T cells, in the presence or absence of varied concentrations of fisetin or other test compounds, were determined by an “in-house” sandwich ELISA immunoassay (15, 131). The coating antibody was anti-human IL- 17A (clone 41809.111; R&D Systems) and the detection antibody was biotinylated polyclonal goat anti-human IL-17A (R&D Systems). The IL-17A ELISA assay sensitivity was <3 pg/ml.

### Ethical statement, *in vivo* study design, and treatment protocols

All animal experiments in this study were approved (Protocol # 18MAY-JCC-01) by the Institutional Animal Care and Use Committee at the University of Louisiana at Monroe (ULM) and conducted in accordance with the guidelines of the ARRIVA and with strict adherence as defined by the NIH guidelines. Female mice (BALB/c, or C57BL/6 J, 5-6 weeks old) were purchased from the Jackson Laboratory (Bar Harbor, ME, USA), housed and acclimatized under pathogen-free conditions at a constant temperature level of 23 ± 2°C, relative humidity of 55-65%, with a 12h light/12h dark cycles at the ULM Animal Resource Facility for one week prior to commencement of experiments. The animals were provided free access to pelleted rodent chow (Harlan/Teklad, Madison, WI, USA) and had access to unlimited amounts of water and feed during the entire study.

Mice dorsal skin hair was shaved/trimmed with an electric trimmer and residual hair roots removed with hair removal cream Nair. All animals were observed closely for signs of inflammation within 48 h prior to experimental procedure. At 7-8 weeks of age, mice received a daily topical dose of 62.5 mg IMQ (5% Aldara cream) on the shaved back skin, and 10 mg IMQ/right ear, and were monitored for signs of skin inflammation every day. The 5% Aldara cream containing IMQ a Toll-like receptor 7/8 ligand and potent immune activator applied topically in mice to generate psoriasiform dermatitis. At the appearance of signs of inflammation after 2-3 days, mice were randomized into 4 groups: control group (no IMQ and vehicle only), IMQ (disease) group, IMQ + Fisetin group (IMQ+Fis), and IMQ + Rapamycin group (IMQ+Rapa) (n = 5-7 mice per group). A daily dosed topical regimen was treatment with fisetin (1 mg/cm^2^ or 50 mg/kg body weight) or rapamycin (0.5 mg/cm^2^ area or 25 mg/kg body weight) 3h before treatment with IMQ. All treatments including fisetin and rapamycin lasted for 9 consecutive days, and control mice were treated similarly with vehicle control, vanicream, while IMQ treatments was given for 11-12 days before mice were euthanized. During the experiments, mice were weighed daily and given warm saline subcutaneously as needed based on calculated weight loss from day 0 of the study.

### Clinical evaluation using pre-clinical photographs

High-resolution photographs (Nikon D7500, Nikon 18-to-80–mm lens; Nikon, Tokyo, Japan; Samsung Galaxy Note9 # SM-N960U1; Samsung Electronics, Suwon, South Korea) were taken at the end of the experiment while mice were under isoflurane anesthesia (VETone Fluriso™, Fluonzo). To compare the size and clinical scores, the photographs were scaled to a millimeter ruler. All mice ears, and back skin were assessed for the severity of the psoriasis-like skin inflammation condition on days 0, and every alternate day, and were objectively scored for erythema, thickening, and scaling using the clinical Psoriasis Area and Severity Index (PASI) scale to assign a score of 0-4 (0, none; 1, mild; 2, moderate; 3, marked; 4, severe). Scoring was performed independently by 4 blinded researchers. Thicknesses of skinfold on the back (on the day of euthanasia) and of the right ear (day 0 and on the day of euthanasia) measured by using an electronic Digital Vernier Calipers (accuracy: ± 0.02 mm, FisherScientific, Pittsburgh, PA, USA). On last experimental day during harvesting (92), all the mice were euthanized and the shaved back and the ear skin area immediately excised and harvested for histological, biochemical, and other analyses.

### Histology, morphometry, and immunostaining analyses of tissue sections

Six millimeters (6 mm) punch biopsy (ear) and sections of skin lesion were fixed in formalin and paraffin-embedded for histological analysis. The remaining skin lesion sections were snap-frozen in liquid nitrogen and stored at -80°C. The formalin fixed paraffin-embedded (FFPE) tissues were cut into 5 μM cross-sections, and were de-paraffinized by incubation in xylene (soaking 2 × 10 min), rehydrated with ethanol (twice in 100% ethanol for 10 min, twice in 95% ethanol for 10 min. twice in 70% ethanol for 10 min, and twice in 50% ethanol, and finally in distilled water for 10 min). Slides were soaked in 1× TBS for 5 min at room temperature before staining. Antigen retrieval was performed by treatment with fresh sodium citrate buffer with 0.05% Tween 20 at pH 6.0, for 30 min at 95-100°C. For histologic staining, tissue sections were stained with Hematoxylin (H) & and Eosin Y (E) solution (Vector Laboratory) and evaluated for gross morphology, epidermal and horny layer thickness and were immunostained for the specified markers were as previously described (139–141). Briefly, endogenous peroxidase was blocked with 3% H_2_O_2_ and the sections were incubated with blocking solution containing 10% normal goat serum (NGS)/0.4% Triton X-100 in PBS for 60 min at room temperature, followed by overnight incubation at 4°C with following antibodies: Rabbit anti-Phospho-Akt (Ser473) (D9E)(1:100), Rabbit anti-Phospho-mTOR (Ser2448) (1:100), PhosphoPlus^®^ p70 S6 Kinase (Thr389, Thr421/Ser424) (1:100) Antibody Kit #9430, Rabbit anti-Phospho-eIF2α (Ser51) (1:100), Rabbit anti-Phospho-4E-BP1 (Thr37/46) (236B4)(1:100), Rabbit anti-peIF2α (1:50), Rabbit anti-STAT3 (1:100), Rabbit anti-CD31 (2H8) (1:100), Rabbit anti-iNOS Monoclonal (4E5) (1:100), and anti-IL-22 (1:50). After three washes, samples were incubated for 2 h with Anti-Rabbit IgG (Goat), HRP-Labeled (1: 600; PerkinElmer NEF812001EA), followed by three washes of 10 min each. ImmPACT^®^ DAB EqV Peroxidase (HRP) Substrate (SK-4103) was used as chromogenic substrate. For counterstaining, a weak solution of haematoxylin was used, and the sections were then dehydrated, mounted with Toluene-based mounting medium (Fisher Diagnostics, Middletown, VA, USA), visualized and analyzed. Slides were visualized on an Olympus IX71 system microscope (Olympus/HuntOptic & Imaging, Inc., Pittsburg, PA, USA), and digital images at 10× to 60× magnification were captured with an Olympus U-CMAD3 attached Olympus DP71 camera (Olympus/HuntOptic & Imaging) linked to a high-resolution computer screen and connected to an Olympus U-RFL-T Mercury Burner. Images were processed by using CellSens dimension software v1.6. The thickness in terms of the area of the entire epidermis and the stratum corneum were determined as earlier described (139, 140).

For immunofluorescence staining, tissue sections were blocked with 10% NGS (Vector Laboratories, Burlingame, CA) or 10% normal donkey serum (Vector Laboratories)/0.4% Triton X-100 in PBS for 1-2 h at room temperature. The following primary antibodies (see Chemicals and Reagent section) were incubated overnight at 4°C including Rabbit Anti-Loricrin antibody (1:100), Mouse anti-F4/80 (1:100), and anti-filaggrin-FITC (1:400), Rabbit anti-CD4 (1:100), Rabbit anti-IL-17 (1:100), mouse anti-mCD45 (1:300), and Rabbit Anti-LC3A/B (D3U4C) (1:400), Rabbit anti-IL1B and mouse anti-FABP5 (1:200), goat anti-human IL-17A, and rat anti-mouse CD45 (#MAB114). After three washes, samples were incubated for 2 h fluorophore-conjugated secondary antibodies, including Texas Red@-X Goat Anti-mouse (1: 1000; #T6390, Invitrogen) and Alexa fluor Goat Anti-rabbit, (1:1000; #A11008, Life Technology), Goat anti-Mouse IgG (H+L) Highly Cross-Adsorbed Secondary Antibody, Alexa Fluor™ Plus 488 (1: 1000; #A32723, ThermoFisher), Goat anti-Rabbit IgG (H+L) Cross-Adsorbed Secondary Antibody, Texas Red-X (1:1000, #T-6391, Thermofisher), followed by three washes of 10 min each. Then, the sections were mounted with the mounting media with 4′,6-diamidino-2- phenylindole (DAPI) (#H-1500, Vector Laboratories). Slides were visualized on an Olympus IX71 system microscope, and digital images at 20× magnification were captured with a DP71 camera connected to an Olympus U-RFL-T Mercury Burner. Images were processed by using CellSens dimension software v1.6 as above.

### Morphometry and quantification

For quantification of epidermal area and thickness of IMQ treated mice, field view micrographs were pieced together using Adobe Photoshop CS4. Pixel values were calibrated to micrometer values, then epidermal areas on histological sections were quantified in binary histological images, generated by manual selection of the epidermis. Photomicrographic image analysis was performed using ImageJ version 1.44 (National Institutes of Health, Bethesda, MD, USA; https://imagej.nih.gov/ij) (142). For the IMQ studies, epidermal thickness was quantified by averaging line measurements of the viable epidermis (without the stratum corneum) in ImageJ software. Quantifications of positive staining cells were conducted using the cell counter and Toolbox plug-in feature in ImageJ, where immune cells, including Ki67+, CD4^+^, CD45^+^, IL-17A^+^ T-cells, and F4/80+ macrophages, were quantified by tracing lines in ImageJ (143, 144).

### Immunocytochemistry and Immunofluorescence staining

HEKa cells, seeded in 6-well Lab-Tek chamber slides (Thermo Scientific, Boston, USA), were pre-treated with fisetin with or without 48-h stimulation with cytokines. After aspiration of the medium, slides were washed with PBS and were fixed with ice-cold 100% methanol for 15 min at -20°C, followed by 3 washes. After permeabilization, cell slides were blocked using 5% NGS in PBS-T for 45 min and incubated overnight at 4°C in a humidified chamber with primary antibodies including Rabbit anti-LC3A/B (1:400) and mouse anti-actin (1:400). For isotype controls, goat anti-rabbit and anti-mouse IgG (DakoCytomation) were used. The slides were washed three times, and then incubated with Texas Red@-X Goat Anti-mouse (1: 1500; #T6390, Invitrogen) and Alexa fluor Goat Anti-rabbit, (1:1500; #A11008, Life Technology) for 2 h in dark. The slides were washed 3× 10 min and then mounted under a coverslip with a drop of *in situ* mounting medium with DAPI (#H-1500, Vector Laboratories). Slides were visualized and fluorescence digital images were obtained/captured with an Olympus IX71 system microscope with an Olympus U-CMAD3 attached DP71 camera (Olympus/HuntOptic & Imaging) linked to a high-resolution computer screen and connected to an Olympus U-RFL-T Mercury Burner. Images at 20× magnification were processed using a CellSens dimension software v1.6. Fluorescence intensity (FI) was quantified in regions of interest (ROI) using ImageJ version 1.44 (NIH, Bethesda, MD, USA). FL intensity was detected as mean brightness value of the ROI. A summary of n = 5-8 image sections in each slide quantified from 2 independent experiments was investigated.

### Mouse ProcartaPlex™ multiplex cytokine/chemokine immunoassay (Affymetrix)

The multiple dysregulated secretion of proinflammatory immune mediator responses in IMQ-induced murine psoriasis-like skin lesions and the role of topical fisetin intervention were examined using a mouse procarta 36-Plex beads-based multiplex ELISArrays multiplex immunoassay technique (Affymetrix/eBioscience) according to the manufacturer’s instruction and as detailed in **Supplementary Methods**.

### Statistical analysis

Statistical analyses were carried out with GraphPad Prism version 9.3 (San Diego, CA, USA), except for ELISA analyses which were performed using the SigmaPlot 11.0 software package (Systat Software, Inc., San Jose, CA, USA). All quantitative data were expressed as means ± SD or SEM, and significant differences were determined by the student t-test or ANOVA with Bonferroni or turkey *post hoc* testing, and *P* values <0.05 were considered significant.

## Data availability statement

The original contributions presented in the study are publicly available. This data can be found here: https://www.ncbi.nlm.nih.gov/geo/query/acc.cgi?acc=GSE217552.

## Author contributions

Conceptualization, SH, KK, SE, and JC.; methodology, SB, SB-M, AW, R-CC, LK, SE, AW, JK and JC; software, TR, ER, and LK**;** validation, TR, SH, JK, SB, SE, and JC; writing - original draft preparation, TR, SH, and JC; writing-review and editing, TR, JK, BN, LK, ER, AW, SE, KK, and SH; visualization, TR, JK, and JC; supervision, BN, SH, JK, KK and JC; project administration, TR and JC; funding acquisition, SH, KK and JC. All authors contributed to the article and approved the submitted version.

## Supplementary material

Supplementary Figure S1 GO and KEGG pathway analysis of differentially expressed genes in different groups shows 20 significantly modulated pathways in bubble plot.

Supplementary Figure S2 Heatmap shows expression of 707 genes in all replicates fisetin group in 2>log2FC in upregulated and downregulated (left). 26 genes were upregulated by cytokine activated keratinocytes compared to the control, while fisetin treatment reversed it towards control. On the other hands, 16 genes were upregulated by fisetin treated cytokine activated keratinocytes shown in right, for all the replicates.

Supplementary Figure S3 Fisetin inhibits the Psoriasis Area and Severity Index in Balb/c mice. **(A)** Schematic overview of preclinical model of mice ear induced with imiquimod showing psoriasis like lesions and reduced psoriasis like dermatitis in treatment groups. **(B)** Photomicrographs of balb/c mice ears of control, IMQ-treated alone and IMQ-co-treatment with topical fisetin and Topical rapamycin. Lesions are clearer in IMQ-treated ears in these Balb/c mice compared to the dark lesions seen in the C57BLJ mice. Average scoring was calculated from each group (n=6) and were compare with other groups.

Supplementary Figure S4 Fisetin inhibits the expression of IL-22, iNOS and CD31 in mice skin. A) Immunohistological staining shows increased expression levels pro-inflammatory cytokine IL-22, cytokine-surge responsive effector iNOS and angiogenesis related marker CD31 in IMQ-induced mice back skin(middle) significantly. Topical application of fisetin reduced all three markers showed in the right column. B) Mean intensity is shown in bar graph as ± SD are shown, paired t test was used to compare values between conditions (n=6). Denotes *P <0.05; **P <0.01 and ***P <0.001. (-) indicates control group.

Supplementary Figure S5 Fisetin normalizes the expression of filaggrin and infiltration of CD4+ T cells in IMQ-induced mice skin. **(A)** Immunohistofluorescence staining of skin tissue section shows increased expression levels of filaggrin (green) and infiltration of CD4+ (red) T-cells in imiquimod induced mice skin, that was significantly lower after fisetin and rapamycin treatment. **(B)** Mean fluorescent intensity (filaggrin) as well as average number counts (CD4+ cells) are shown in bar graph as mean±SD, paired t test was used to compare 29 values between conditions n=6 for each group). Denotes *P <0.05; and **P <0.01 ***P 30 <0.001. (ns) indicates not significant.

Supplementary Figure S6 Fisetin inhibits the IL-1β expression in mice skin. **(A)** Immunohistofluorescence staining of skin tissue section shows increased expression levels of IL-1β (green) and counterstained by DAPI (blue) in imiquimod induced mice skin, that was significantly lower after fisetin and rapamycin treatment. **(B)** Mean fluorescent intensity IL-1β is shown in bar graph as Means ± SE are shown, mice per group was n=6, paired t test was used to compare values between conditions. Denotes *P <0.05; and **P <0.01 ***P <0.001. (ns) indicates not significant.

Supplementary Figure S7 Fisetin treatment reduces the serum LDL to HDL ratio compared to imiquimod treated mouse group.

Supplementary Table S2 List of immune mediators that were not markedly modulated upon treatment of IMQ-induced mice skin lesions with fisetin or rapamycin. Each group of mice had 6 mice (n=6).
