# Supplementary figures and images for: Dual targeting of mTOR/IL-17A and autophagy by fisetin alleviates psoriasis-like skin inflammation
Source: Front Immunol. 2023 Jan 18;13:1075804. doi: 10.3389/fimmu.2022.1075804 (PMC9889994; doi:10.3389/fimmu.2022.1075804)

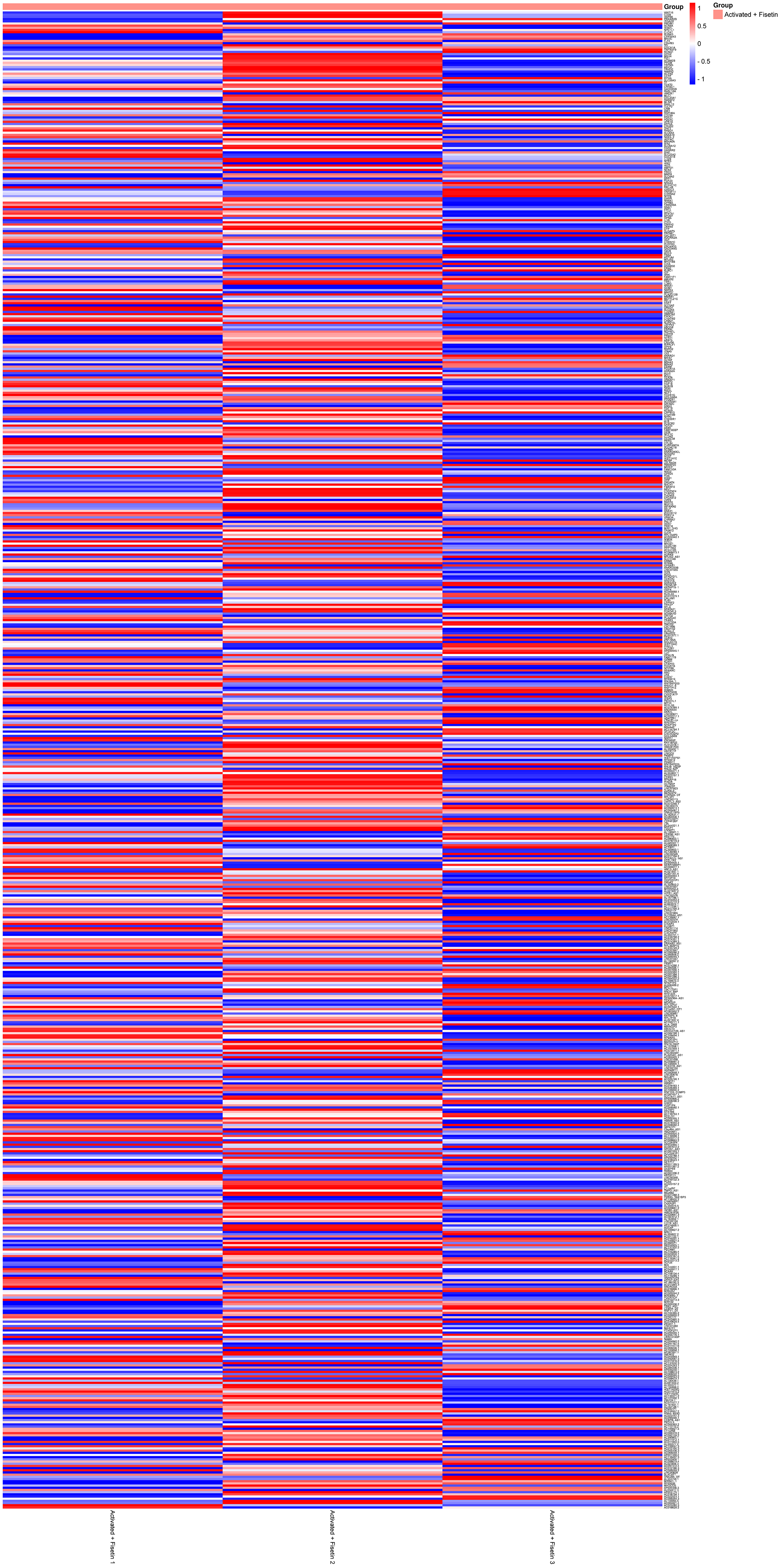

Supplement: Supplementary file 2 [file Image_8.tiff]

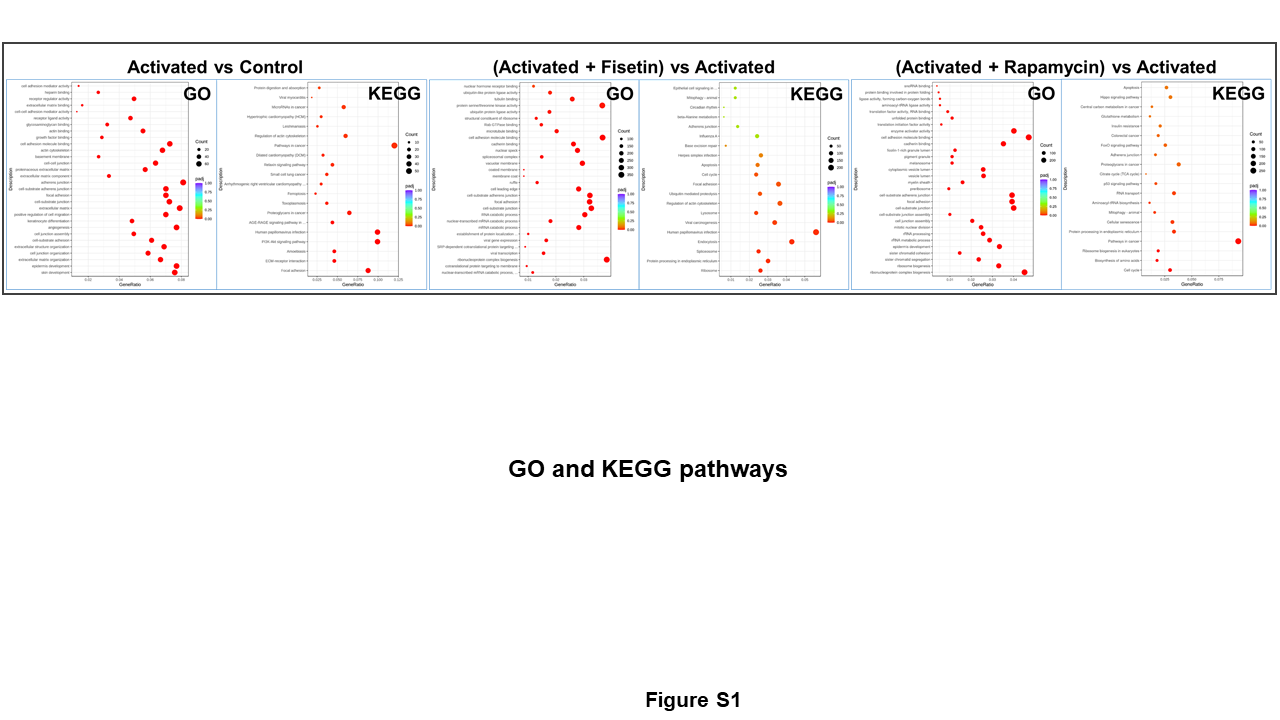

Supplement: Supplementary Figure S1 — GO and KEGG pathway analysis of differentially expressed genes in different groups shows 20 significantly modulated pathways in bubble plot. [file Image_1.tif]

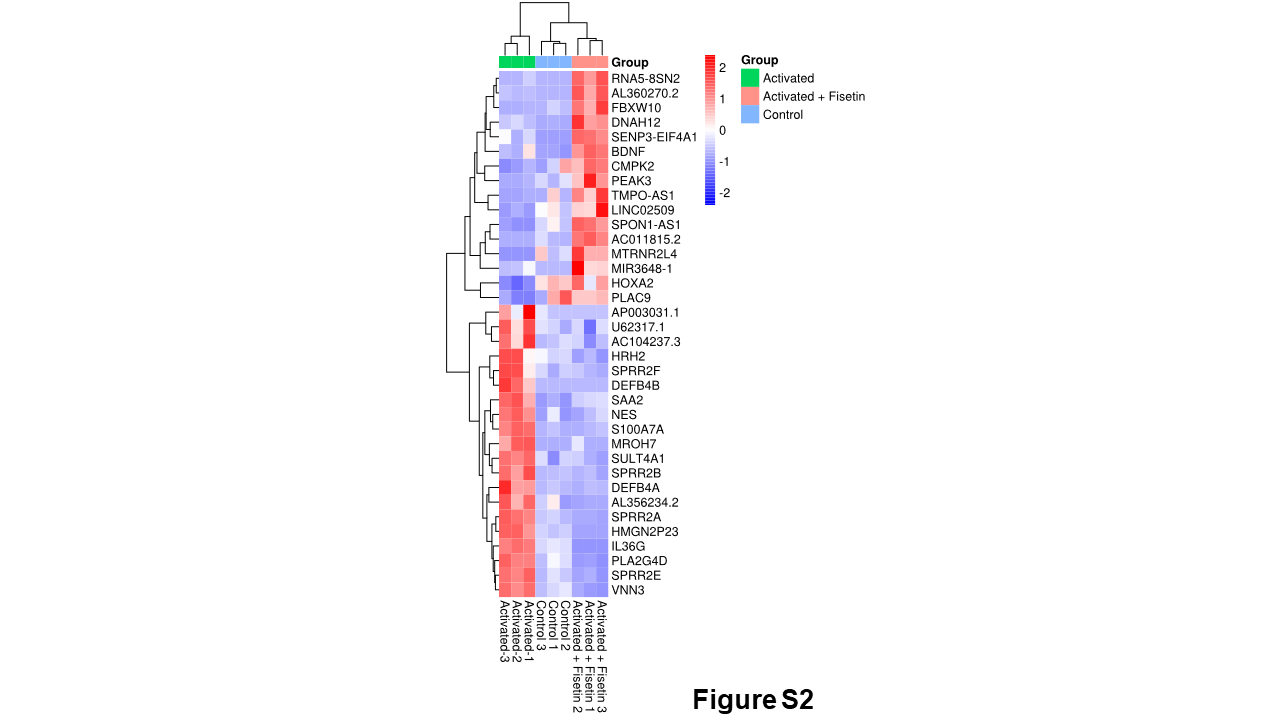

Supplement: Supplementary Figure S2 — Heatmap shows expression of 707 genes in all replicates fisetin group in 2>log2FC in upregulated and downregulated (left). 26 genes were upregulated by cytokine activated keratinocytes compared to the control, while fisetin treatment reversed it towards control. On the other hands, 16 genes were upregulated by fisetin treated cytokine activated keratinocytes shown in right, for all the replicates. [file Image_2.tif]

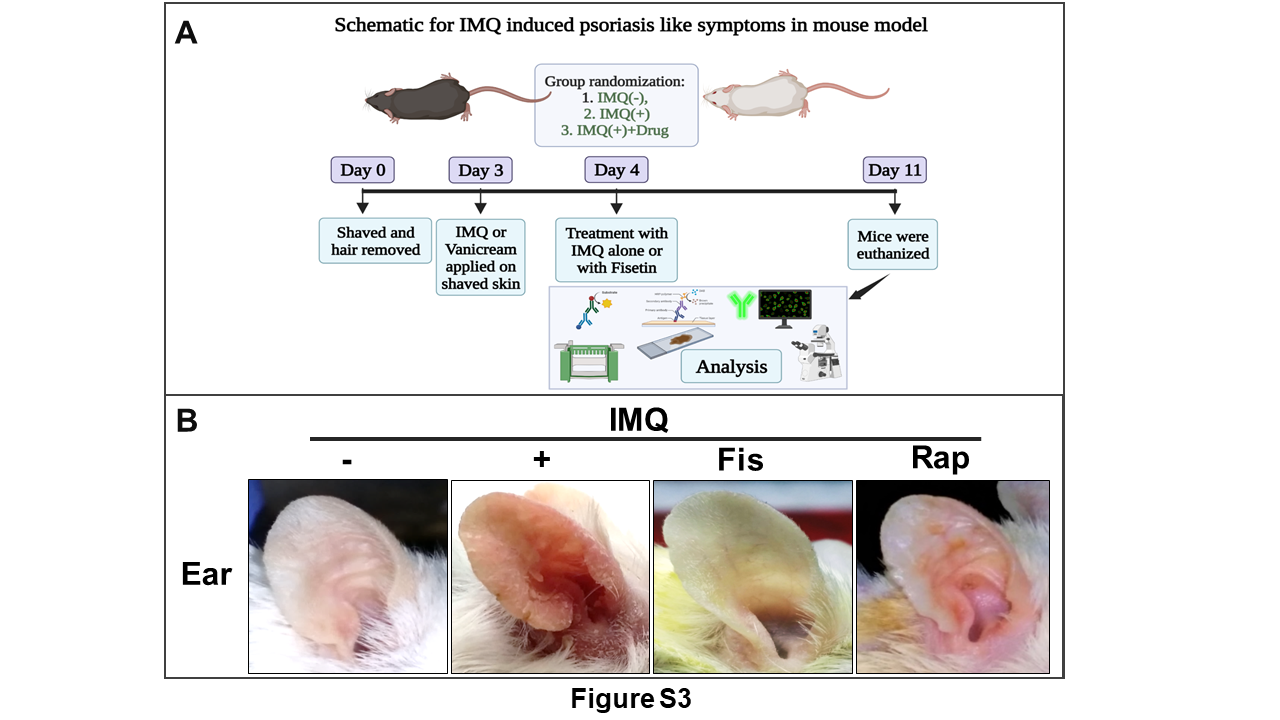

Supplement: Supplementary Figure S3 — Fisetin inhibits the Psoriasis Area and Severity Index in Balb/c mice. (A) Schematic overview of preclinical model of mice ear induced with imiquimod showing psoriasis like lesions and reduced psoriasis like dermatitis in treatment groups. (B) Photomicrographs of balb/c mice ears of control, IMQ-treated alone and IMQ-co-treatment with topical fisetin and Topical rapamycin. Lesions are clearer in IMQ-treated ears in these Balb/c mice compared to the dark lesions seen in the C57BLJ mice. Average scoring was calculated from each group (n=6) and were compare with other groups. [file Image_3.tif]

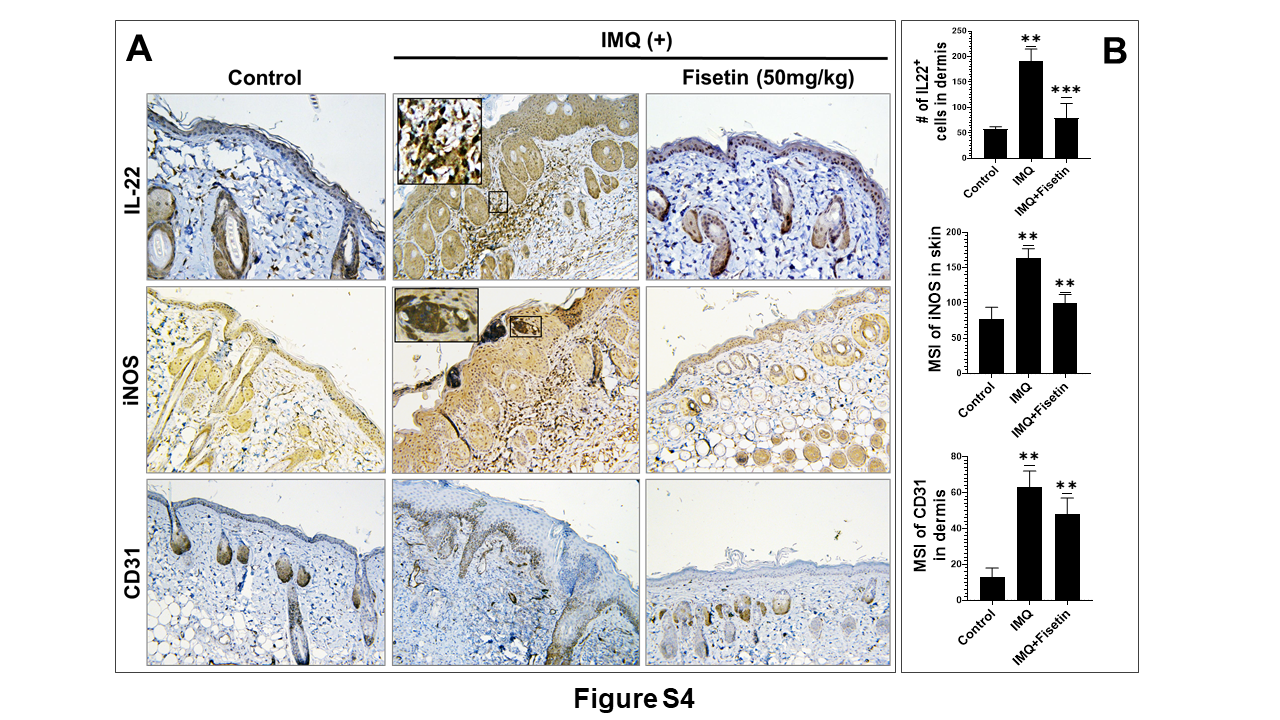

Supplement: Supplementary Figure S4 — Fisetin inhibits the expression of IL-22, iNOS and CD31 in mice skin. A) Immunohistological staining shows increased expression levels pro-inflammatory cytokine IL-22, cytokine-surge responsive effector iNOS and angiogenesis related marker CD31 in IMQ-induced mice back skin(middle) significantly. Topical application of fisetin reduced all three markers showed in the right column. B) Mean intensity is shown in bar graph as ± SD are shown, paired t test was used to compare values between conditions (n=6). Denotes *P <0.05; **P <0.01 and ***P <0.001. (-) indicates control group. [file Image_4.tif]

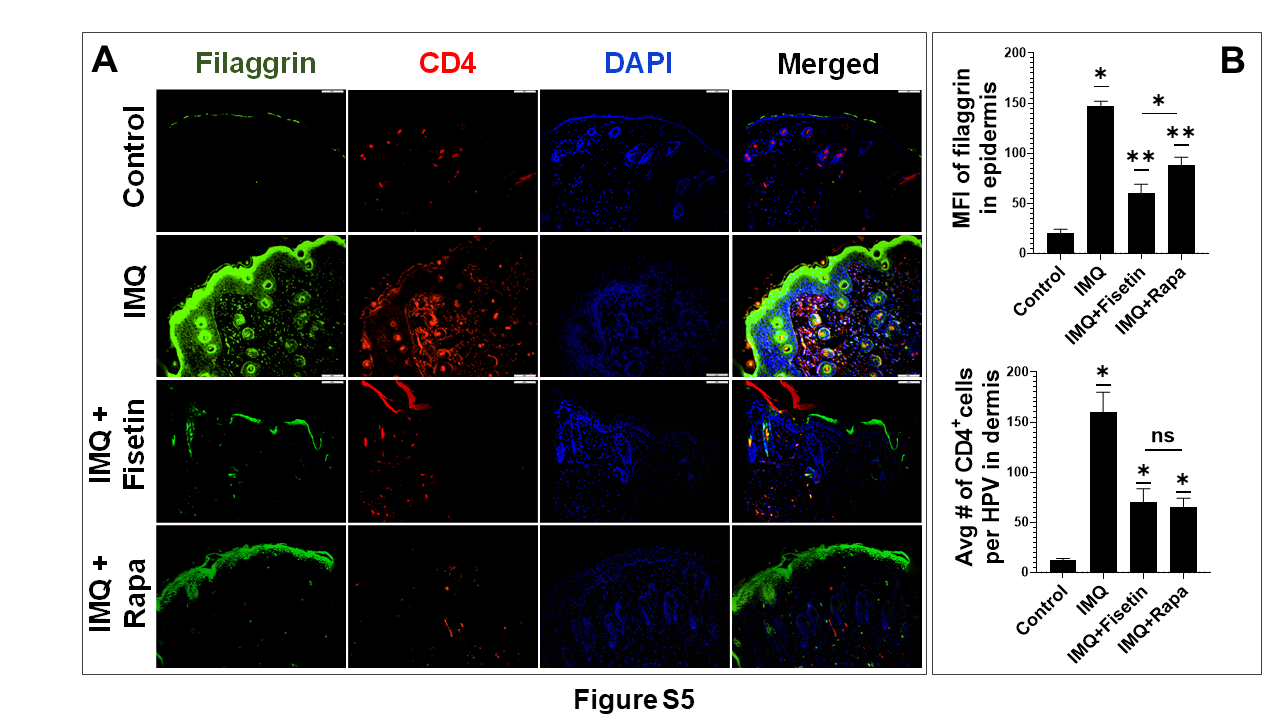

Supplement: Supplementary Figure S5 — Fisetin normalizes the expression of filaggrin and infiltration of CD4+ T cells in IMQ-induced mice skin. (A) Immunohistofluorescence staining of skin tissue section shows increased expression levels of filaggrin (green) and infiltration of CD4+ (red) T-cells in imiquimod induced mice skin, that was significantly lower after fisetin and rapamycin treatment. (B) Mean fluorescent intensity (filaggrin) as well as average number counts (CD4+ cells) are shown in bar graph as mean±SD, paired t test was used to compare 29 values between conditions n=6 for each group). Denotes *P <0.05; and **P <0.01 ***P 30 <0.001. (ns) indicates not significant. [file Image_5.tif]

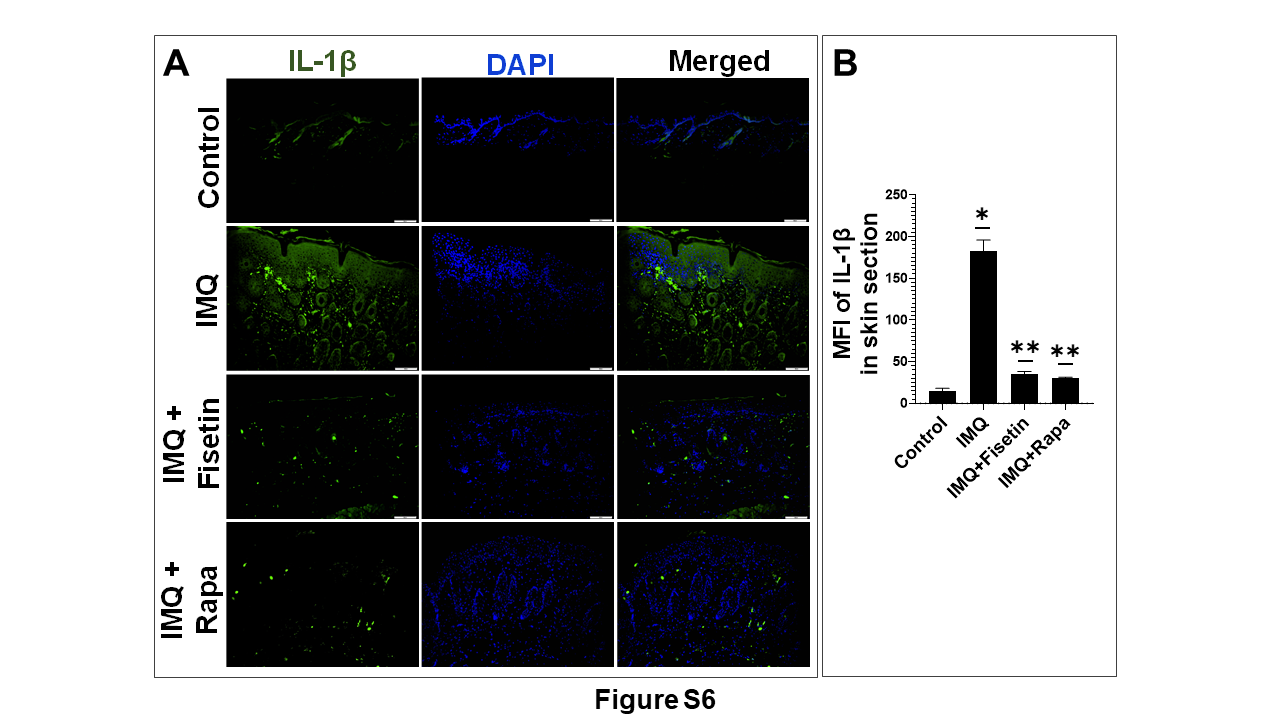

Supplement: Supplementary Figure S6 — Fisetin inhibits the IL-1β expression in mice skin. (A) Immunohistofluorescence staining of skin tissue section shows increased expression levels of IL-1β (green) and counterstained by DAPI (blue) in imiquimod induced mice skin, that was significantly lower after fisetin and rapamycin treatment. (B) Mean fluorescent intensity IL-1β is shown in bar graph as Means ± SE are shown, mice per group was n=6, paired t test was used to compare values between conditions. Denotes *P <0.05; and **P <0.01 ***P <0.001. (ns) indicates not significant. [file Image_6.tif]

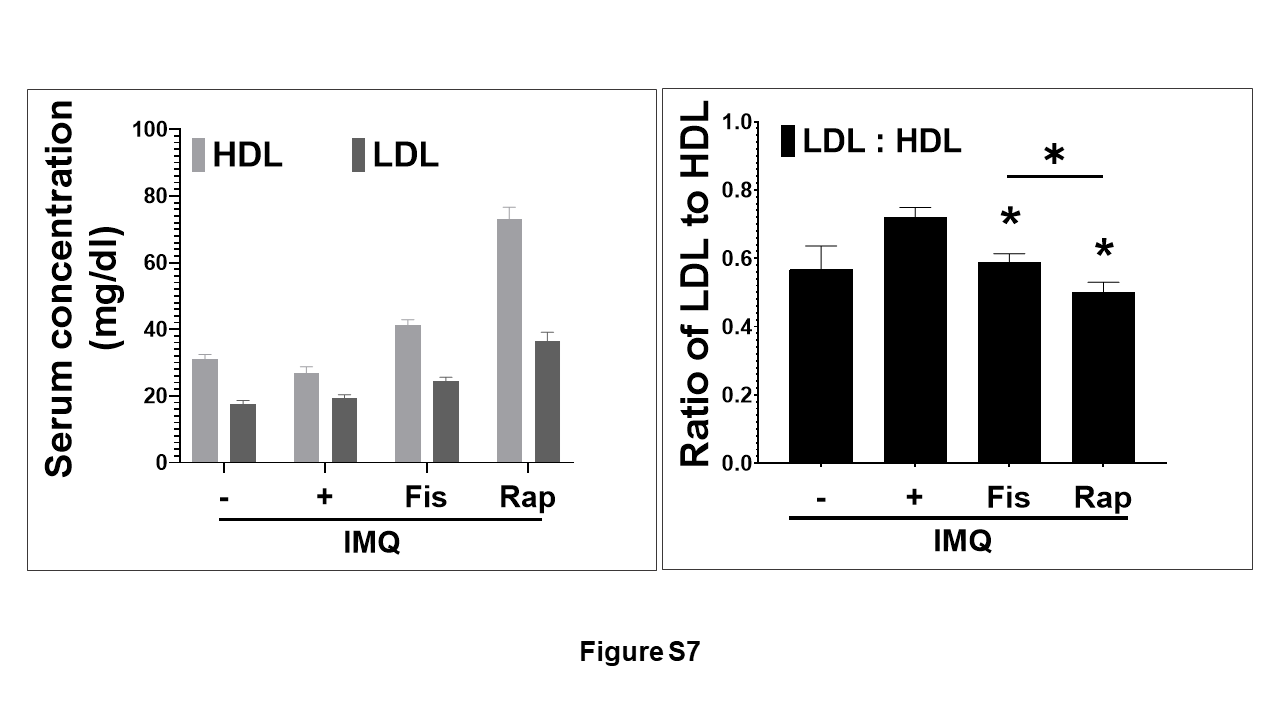

Supplement: Supplementary Figure S7 — Fisetin treatment reduces the serum LDL to HDL ratio compared to imiquimod treated mouse group. [file Image_7.tif]
